# Rules of engagement: Determinants of chemokine receptor activation and selectivity by CCL27 and CCL28

**DOI:** 10.1016/j.jbc.2025.110736

**Published:** 2025-09-18

**Authors:** Mian Huang, Aura F. Celniker, Rezvan Chitsazi, Douglas P. Dyer, Ariane L. Jansma, Irina Kufareva, Catherina L. Salanga, Tracy M. Handel

**Affiliations:** Skaggs School of Pharmacy and Pharmaceutical Sciences, University of California, San Diego, La Jolla, California, USA

**Keywords:** G protein-coupled receptor (GPCR), cell migration, glycosaminoglycan, structure-function, CCL27, CCL28, CC chemokine receptor type 3 (CCR3), CC chemokine receptor type 10 (CCR10), superagonist, AI-powered molecular modeling, AI-based interaction scoring

## Abstract

The distinct functional roles of chemokines CCL27 and CCL28 in epithelial immunity of skin and mucosal tissues, respectively, are coordinated by their shared receptor, CCR10, and the CCL28-specific receptor, CCR3. To identify determinants of receptor activation, internalization and binding specificity, we conducted structure-function studies focused on the N termini of these two chemokines. Deletion of two N-terminal residues of CCL27 resulted in a CCR10 antagonist, highlighting the critical roles of these residues in driving receptor pharmacology. Extension with a Phe produced a CCR10 superagonist by occupying a unique subpocket in the receptor. Swapping the CCL28 N terminus onto the CCL27 globular domain (NT28-CCL27) also resulted in a superagonist of CCR10, but the opposite swap (NT27-CCL28) showed equivalent or reduced activity compared to WT CCL28, indicating that the CCL28 N terminus is a stronger driver of CCR10 signaling. The effects of these mutations were rationalized by AlphaFold models of the CCR10 complexes. Modeling also revealed that the reduced size of the binding pocket, and more basic nature of the N terminus and extracellular loops of CCR3 compared to CCR10, contribute to its specificity for CCL28 while CCR10 accommodates both ligands. The basic nature of CCL28 also contributes to its high affinity for glycosaminoglycans and is likely important for its retention in mucosal tissues. These data illustrate how the modular nature of these chemokines enables their overlapping but nonredundant functions, and how this modularity can be exploited to produce engineered chemokines for probing or targeting CCR10 in disease.

Chemokines and their receptors belong to a large family of chemoattractant proteins that control cell movement and positioning during development and homeostatic processes, as well as during inflammatory responses to infection and other physiological insults (1, 2). As central mediators of inflammation, they have been pursued as potential therapeutic targets in numerous inflammatory, autoimmune and infectious diseases (3, 4, 5). CC chemokines, CCL27 and CCL28, both ligands of CC chemokine receptor 10 (CCR10), are critical regulators of epithelial immunity, but play functionally unique roles as suggested by their distinct expression patterns. CCL27 is constitutively expressed by skin keratinocytes (6) and CCL28 by epithelial cells of various mucosal tissues including the gastroinstestinal tract, salivary glands, mammary glands, nasal epithelial cells, and lung (7, 8, 9). CCR10 is expressed on skin homing T cells, including cutaneous lymphocyte-associated antigen positive memory T cells (6, 10, 11), T regulatory cells (12), as well as melanocytes, dermal fibroblasts, and dermal microvascular endothelial cells (13) that respond to constitutively produced or upregulated CCL27. CCR10 is also expressed by mucosal IgA antibody-producing plasma blasts and plasma cells that respond to CCL28 (14). The potential of CCR10 as a therapeutic target is suggested by its role in T cell–mediated skin inflammation such as psoriasis (6) and atopic or allergic-contact dermatitis (10, 15), in addition to its expression on lung epithelial cells in humanized mouse models of idiopathic pulmonary fibrosis (16). Additionally, CCR10 contributes to various cancers both through its expression directly on malignant cells and by its expression on cells in the tumor microenvironment. For example, engagement of CCL27 by CCR10 expressed on skin melanoma cells promotes immune evasion (17), whereas its expression on T regulatory cells and the CCL28-mediated recruitment of those cells into the tumor microenvironment of ovarian and liver cancer promotes tumor growth through the creation of an immunosuppressive microenvironment (18, 19). Thus, blocking CCR10 may be a useful approach for skin inflammatory diseases, idiopathic pulmonary fibrosis, and enhancing the efficacy of cancer therapies (20, 21).

In addition to CCR10, CCL28 also binds CC chemokine receptor 3 (CCR3), which is expressed on CD4+ T cells in nasal mucosa (22), as well as eosinophils (8, 23), basophils, and neutrophils (24) following infection. CCR3 is a promiscuous receptor with numerous chemokine ligands (*e.g.*, CCL5, CCL7, CCL11, CCL13, CCL15, CCL24, CCL26, and CCL28 (25)), although with the exception of CCL28, most exhibit little to no expression on normal nasal mucosa (22), suggesting a specific role of CCL28 and CCR3 homing of CD4+ T cells to the upper airways. CCR3 is implicated in numerous allergic diseases including asthma, eosinophilic esophagitis, and atopic dermatitis (26, 27) making the CCR3 axis another attractive pharmaceutical target.

Given their importance in both normal physiology and disease, surprisingly little is known about the structure-function details of CCL27 and CCL28, apart from NMR structures (28, 29). Thus, to understand the molecular interactions of CCL27 and CCL28 with their receptors, we undertook a mutagenesis study focusing on the chemokine N-terminal residues, which are key determinants of receptor pharmacology. Studies of effects on signaling and internalization revealed variants of CCL27 that are superagonists of CCR10. AlphaFold (AF) models of WT and mutant chemokines in complex with CCR10 and CCR3 rationalized the functional consequences of the mutations, as well as the specificity of CCL27 for CCR10 and CCL28 for CCR10 and CCR3. Our functional data and computational models also provide a starting point for further optimization of CCL27 superagonists that may have utility as adjuvants for vaccine development (30, 31). In addition to signaling receptors, chemokines interact with glycosaminoglycans (GAGs) (32, 33), which contributes to their localization on cell surfaces and the extracellular matrix (34). Here, we demonstrate that CCL27 and CCL28 exhibit markedly different GAG-binding propensities that correlate with their accumulation on cells. Along with their distinct expression profiles, these findings underscore the nonredundant roles of the two chemokines, despite sharing CCR10 as a common receptor.

## Results

### Rationale for the present study

**Figure 1:**
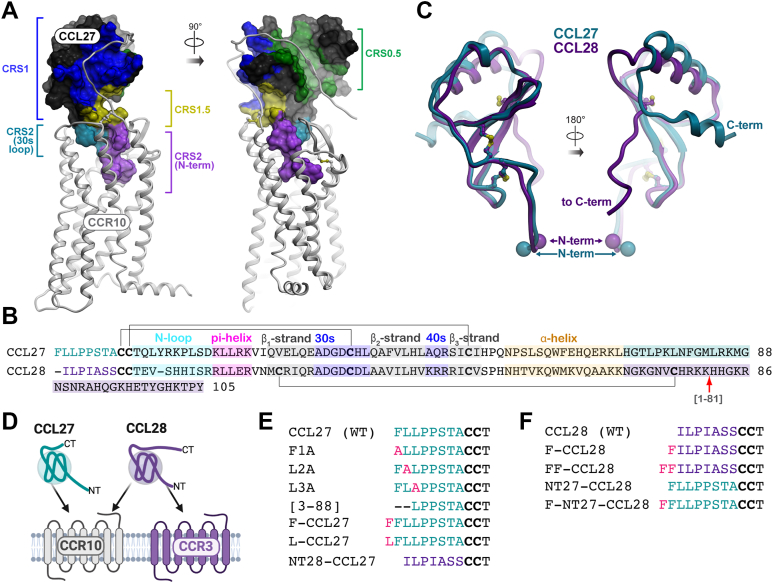
**Study rationale for the CCL27/CCL28 structure-function investigation.***A*, general architecture of receptor–chemokine complexes, illustrated by a complex of CCR10 and CCL27. The complex is shown in two orientations; the receptor is shown as *white ribbons* and the chemokine as a *surface mesh*, with key interaction epitopes color-coded and labeled. *B*, sequence alignment of CCL27 and CCL28 where the N-terminal residues are colored *teal* and *purple*, respectively, and characteristic chemokine features labeled accordingly. Disulfide bonds are indicated by *black lines*, the CCL28 C-terminal truncation mutant is indicated by a *red arrow* (residue 81 of CCL28) and the extended C-terminal domains are highlighted. *C*, overlay of structural models of CCL27 (*teal*) and CCL28 (*purple*). Chemokines are shown as *ribbons*, in two orientations, disulfide bonds as *sticks*, and the N and C termini are labeled. *D*, *cartoon* depicting CCL27 specificity for CCR10 and CCL28 for CCR3 and CCR10. *E*, sequences of WT and N terminally modified mutants of CCL27 and (*F*) CCL28. Disulfide bonded Cys residues are *bolded*. CCR, CC chemokine receptor.

All chemokines share a globally similar architecture when engaging their receptors, whereby the chemokine globular domain rests on the extracellular face of the receptor and interacts with the receptor N terminus (regions referred to as chemokine recognition sites 1 and 0.5, CRS1 and CRS0.5) and extracellular loops (ECLs), while the chemokine N terminus reaches into the transmembrane (TM) receptor binding pocket (CRS2) and promotes activation through rearrangements of the TM helices (35, 36, 37) (Fig. 1*A*). Accordingly, the N-termini of most chemokines drive the pharmacological responses of their receptors (38, 39, 40, 41) (Fig. 1*A*). Extension of some chemokines by even a single amino acid can profoundly increase or decrease efficacy (41, 42, 43). Truncation of chemokine N termini can be activating or deactivating with effects on receptor binding affinity, signaling and trafficking (41, 44), and proteolytic N-terminal processing represents a natural mechanism for regulating chemokine activity (45, 46, 47).

CCL27 and CCL28 share 36% sequence homology (Fig. 1*B*) and the global architecture typical for chemokines (Fig. 1*C*), but also have some unique features. The N terminus of CCL28 is only 7 amino acids long, compared to 8 amino acids in CCL27, with the amino acid sequence similarity between the two chemokine N termini modest at best (Fig. 1*B*). In addition to the C-terminal α-helix present in all chemokines, the C-terminal amino acids (81, 82, 83, 84, 85, 86, 87, 88) of CCL27 are predicted to form a second helix, folding next to the first one (Fig. 1*C*). By contrast, the strongly basic 105 amino acid C terminus of CCL28 is predicted to be unstructured, with its base stapled to the β1-strand of the chemokine *via* a third disulfide bond, absent in most chemokines (Fig. 1, *B* and *C*).

Considering these differences, we sought to understand the molecular features that allow both chemokines to activate CCR10 but only CCL28 to activate CCR3 (Fig. 1*D*). To this end, we generated a number of N-terminal modifications of CCL27 (Fig. 1*E*), including a chimeric chemokine with the N terminus of CCL28, and a set of reciprocal mutants of CCL28 (Fig. 1*F*). We then characterized the effects of these modifications on receptor signaling and internalization, and CCR10-*versus*-CCR3 specificity.

### N-terminal mutations of CCL27 produce CCR10 antagonists

**Figure 2:**
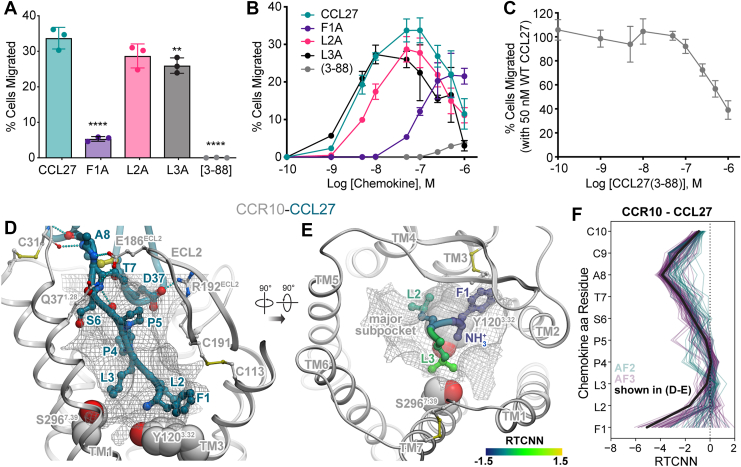
**Identification of N terminally modified CCL27 mutants that function as CCR10 antagonists.***A*, cell migration of WT CCL27 and N-terminal mutants with CCR10-expressing L1.2 cells at 50 nM (average concentration for maximal migration of WT CCL27); data shown are representative (mean ± SD) of three independent experiments performed in technical triplicates. Statistical analysis was performed by ordinary one-way ANOVA with *post hoc* Dunnett’s multiple comparisons test (∗∗∗∗*p* < 0.0001; ∗∗*p* < 0.01; CCL27: L2A, *p* = 0.0673). *B*, migration across a broad chemokine concentration range (0.1 nM to 1 μM), plotted as the percent of cells migrated relative to maximal migration (*i.e.*, no filter). Shown are representative data (mean ± SD) of three independent experiments performed in technical triplicates. *C*, competition chemotaxis binding assay of CCL27 with the N-terminal truncation mutant [3-88]. WT CCL27 (50 nM) was incubated with increasing amounts of [3-88]-CCL27 and the percent cell migration was compared after a 2 h incubation period at 37 °C. Shown are representative data (mean ± SD) of three independent experiments performed in technical triplicates. *D*, structural model of WT CCL27 (*teal ribbon and sticks*) in complex with CCR10 (*gray ribbon*). The optimal ligand atom placement surface in the orthosteric pocket of the receptor is shown as a *gray mesh*. The complex is viewed along the plane of the membrane in the TM2-to-TM5 direction; TM2 is hidden for clarity. Receptor residues Tyr120(3.32) and Ser296(7.39) are shown in *gray spheres*. *E*, the model from (*D*) is viewed perpendicular to the plane of the membrane from the extracellular side; chemokine residues 1 to 3 are shown as *ribbon* and *sticks*; color represents per-atom RTCNN scores aggregated to residue backbones (for *ribbons* and *backbone sticks*); and side chains (for side chain sticks). *F*, RTCNN scores of N-terminal residues of CCL27 across the ensembles of AF2 and AF3 structural models of the CCR10–CCL27 complex. AF, AlphaFold; CCR, CC chemokine receptor; RTCNN, Radial and Topological Neural Network; TM, transmembrane.

As a starting point, three N-terminal Ala mutants of CCL27 (F1A-, L2A- and L3A-CCL27) and an N-terminal truncation mutant ([3-88]-CCL27) (Fig. 1*E*) were generated and characterized in several functional assays. In transwell migration assays with murine L1.2 pre-B cells stably expressing human CCR10, 50 nM L2A and L3A showed an ∼15 to 20% reduction in migration compared to WT CCL27, while F1A showed a reduction of ∼85%, and [3-88]-CCL27 was unable to induce cell migration (Fig. 2*A*). Full dose–response curves revealed an approximately 35% reduction in efficacy and 10-fold reduction in potency for F1A-CCL27 relative to WT CCL27. The truncation mutant [3-88]-CCL27 was an extremely low-efficacy partial agonist with no ability to induce cell migration below 1 μM chemokine (Fig. 2*B*), and as such was able to block cell migration to WT CCL27 (Fig. 2*C*). These data indicate that Phe1 and Leu2, and to a lesser extent Leu3, are required for activation of CCR10 and that [3-88]-CCL27 retains the ability to bind CCR10 and can antagonize the response to WT CCL27.

To understand the structural basis for the observed differences, we generated AF2 and AF3 models of CCL27 bound to CCR10 (Figs. 2, *D*, *E*, S1 and S2, Supp. Data 1–3). In the highest confidence models, residues Phe1, Leu2, and Leu3 of the chemokine directly interact with Tyr120(3.32) and Ser296(7.39) at the bottom of the orthosteric binding pocket of the receptor (numbers in parentheses are Ballesteros–Weinstein indices, a universal residue notation for class A G protein–coupled receptor residues (48)). These pocket residues are known to be involved in the activation of multiple homologous receptors (49, 50, 51, 52, 53). Phe1 of CCL27 favorably packs under the ECL2 and the conserved disulfide bridge Cys113(3.25)-Cys191(ECL2) of the receptor (Fig. 2*D*). To estimate the relative contribution of chemokine residues to the formation of the active-state complex and thus receptor signaling, we used the Radial and Topological Neural Network (RTCNN), a physics-aware artificial intelligence function for scoring protein–ligand interactions (54, 55, 56, 57). This analysis suggested that among the first three residues of CCL27, the biggest contribution is from Phe1 whereas the role of Leu2 is smaller and that of Leu3 is minor (Fig. 2, *E* and *F*), consistent with the impact of their mutations on CCL27-induced migration (Fig. 2, *A* and *B*). RTCNN scores also emphasized the role of the proximal N terminus of CCL27 (Fig. 2*F*) that engages in numerous favorable hydrogen-bonding interactions with the proximal N terminus and ECL2 of the receptor (Fig. 2*D*). These data explain why Phe1 and Leu2 are the primary determinants of CCR10 activation and why [3-88]-CCL27, which lacks these two amino acids, maintains affinity for the receptor and acts as a receptor antagonist.

### A phenylalanine addition to the N terminus of CCL27 produces a CCR10 superagonist

**Figure 3:**
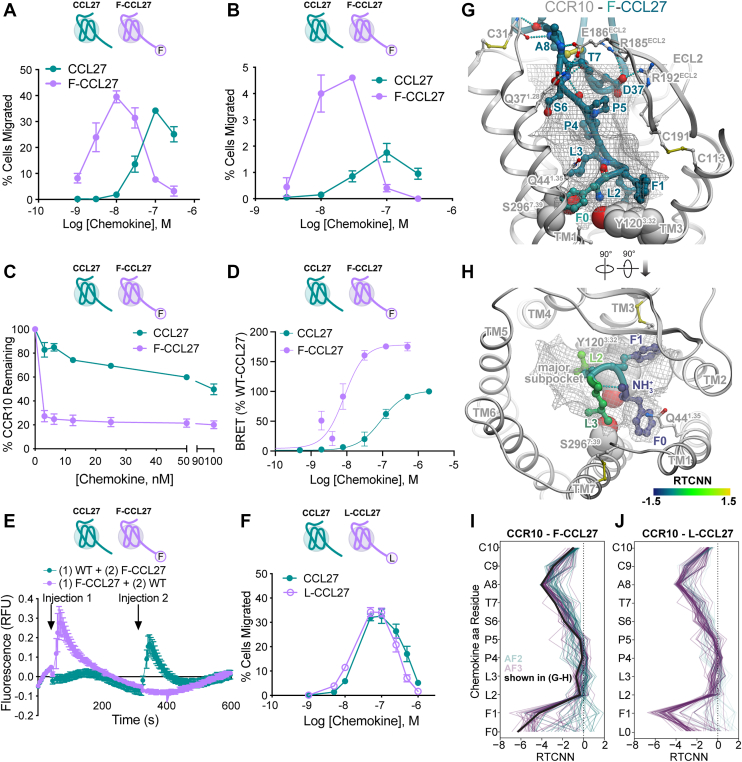
**Discovery of the CCR10 superagonist, F-CCL27.***A*, cell migration analysis of CCL27 WT and F-CCL27 with L1.2/CCR10 cells across varying chemokine concentrations. Results shown are representative data (mean ± SD) of independent experiments (n = 6) performed in technical duplicates or triplicates. *B*, transendothelial cell migration of WT CCL27 and F-CCL27 with L1.2/CCR10 cells across varying chemokine concentrations with data plotted as the percent of cells migrated after a 2 h incubation at 37 °C. Shown are representative data (mean ± SD) of independent experiments (n = 3) performed in technical duplicates. *C*, WT or F-CCL27–mediated internalization of L1.2/CCR10 cells. Cells were treated with varying concentrations of chemokine, incubated at 37 °C for 45 min, and then receptor levels were detected and plotted as the percent of receptor remaining compared to cells treated with no chemokine. Results shown are the mean ± SEM of single measurements obtained for each concentration combined from at least three independent experiments (n = 3–4). *D*, β-arrestin recruitment by BRET. Cells were transiently transfected with CCR10-RlucII and βarr2-GFP10 and then stimulated with varying concentrations of chemokine. The BRET ratios are shown as percentage of WT-CCL27. Results shown are the mean ± SD of independent experiments (n = 4) performed in technical duplicates or triplicates. *E*, the higher potency of F-CCL27 than WT CCL27 as measured by calcium flux using L1.2/CCR10 cells. Cells were treated with an initial dose of 20 nM WT or F-CCL27 (injection 1), followed by a second addition of 20 nM F-CCL27 and WT CCL27 (injection 2), respectively, after the 75th reading cycle (at approximately 320 s). Note that WT CCL27 produces a calcium response at higher concentrations, consistent with a prior study (13), 50 nM in our hands), but 20 nM was chosen in order to observe a signal in response to a second injection of chemokine. Shown are representative data (mean ± SD) of independently performed experiments (n = 3) performed in technical triplicates. *F*, cell migration analysis of CCL27 WT and L-CCL27 with L1.2/CCR10 cells across varying chemokine concentrations plotted as the percent of cells migrated after a 2 h incubation at 37 °C. Shown are representative data (mean ± SD) of independent experiments (n = 3) performed in technical triplicates. *G*, structural model of F-CCL27 (*teal ribbon and sticks*) in complex with CCR10 (*gray ribbon*). The optimal ligand atom placement surface in the orthosteric pocket of the receptor is shown as *gray mesh*. The complex is viewed along the plane of the membrane in the TM2-to-TM5 direction; TM2 is hidden for clarity. Receptor residues Tyr120(3.32), F216(5.47), Y263(6.51), L267(6.55), and Ser296(7.39) are shown as *gray spheres*. *H*, the model from (*G*) is viewed perpendicular to the plane of the membrane from the extracellular side; chemokine residues 0 to 3 are shown as *ribbon* and *sticks*; color represents per-atom RTCNN scores aggregated to residue backbones (for *ribbons* and *backbone sticks*) and side chains (for side chain sticks). *I*-*J*, RTCNN scores of N-terminal residues of F-CCL27 (*I*) and L-CCL27 (*J*) across the ensembles of AF2 and AF3 structural models of CCR10 complexes with these two chemokines. BRET, bioluminescence resonance energy transfer; CCR, CC chemokine receptor; RTCNN, Radial and Topological Neural Network; TM, transmembrane.

We next investigated the impact of adding residues to the N termini of CCL27 and CCL28 since such additions can also modulate signaling, sometimes resulting in useful variants with superagonist or antagonist properties (41, 42, 43, 58) and potential therapeutic value (41, 42, 43). Because these modifications often involve bulky groups, we extended CCL27 with an N-terminal Phe to produce F-CCL27 (Fig. 1*E*) and observed markedly enhanced agonist activity in multiple functional assays (Fig. 3, *A*–*E*). In a bare filter chemotaxis assay involving CCR10-expressing L1.2 cells, the efficacy and potency of F-CCL27 were 1.1- and 10-fold higher, respectively, than those of WT CCL27 (Fig. 3*A*). F-CCL27 also exhibited a 2.7-fold higher efficacy and 3-fold greater potency than WT CCL27 in promoting transendothelial cell migration (Fig. 3*B*); the difference in efficacy was particularly striking compared to the bare filter migration assay (Fig. 3, *A versus B*).

In addition to its effect on G protein–mediated signaling responses, we also investigated whether the addition of the N-terminal Phe impacted the ability of the receptor to internalize following chemokine stimulation using flow cytometry (Fig. 3*C*). F-CCL27 internalized CCR10 much more effectively than WT CCL27, with only ∼30% of the receptor remaining on the cell surface after 45 min compared to ∼80% after stimulation with WT CCL27 at the lowest concentration tested (3.1 nM) (Fig. 3*C*). Since internalization is often a β-arrestin–mediated process (59), we also used a bioluminescence resonance energy transfer (BRET)-based association assay to monitor β-arrestin recruitment to the receptor (60) with CCR10-RlucII and GFP10-tagged β-arrestin2 (βarr2-GFP10) as BRET donor-acceptor pairs (Fig. 3*D*, Table S1). Compared to WT-CCL27, which promoted β-arrestin recruitment to CCR10 with an EC_50_ of 89 nM, F-CCL27 recruited β-arrestin with an EC_50_ of 19 nM; the Emax of F-CCL27 was 1.8-fold that of WT-CCL27 (Fig. 3*D*, Table S1). The higher potency of F-CCL27 than WT CCL27 was also demonstrated in a calcium flux assay. Although WT CCL27 induces a calcium response at 50 nM chemokine, consistent with a prior study (13), it exhibited little to no signal at 20 nM (Figs. 3*E* and S3), while F-CCL27 produced a robust response. Notably, cells initially stimulated with F-CCL27 showed no response to a subsequent stimulation by WT CCL27, whereas cells initially stimulated with WT CCL27 produced a secondary response to F-CCL27, almost as large as the initial response with F-CCL27 (Figs. 3*E* and S3). This suggests that F-CCL27 fully occupied (and possibly desensitized) CCR10 after the first stimulation, while this was not the case for WT CCL27.

By contrast with Phe0, the addition of a N-terminal Leu0 (L-CCL27) showed little to no change compared to WT CCL27 in a bare filter chemotaxis assay (Fig. 3*F*), indicating the specificity of the Phe addition for enhanced activation of CCR10. Taken together, the more potent responses of F-CCL27 with respect to receptor activation, desensitization, and internalization suggest that it is a superagonist. On the other hand, analogous N-terminal modifications in CCL28 (F-CCL28 and FF-CCL28, Fig. 1*F*) had little to no effect on CCR10-mediated migration compared to the WT chemokine in a bare filter chemotaxis assay (Fig. S4*A*), suggesting marked differences in how these ligands activate CCR10.

To investigate the structural mechanism underlying F-CCL27 superagonism, we turned our attention to AF2 and AF3 models of CCR10 bound to F-CCL27 (Figs. 3, *G*, *H*, S1 and S2, Supp. Data 1–3). Compared to AF2, AF3 generated predictions with much higher confidence and conformational consistency (Figs. S1 and S2, Supp. Data 1–3). Across the majority of AF3 models, F-CCL27 shares most of the WT CCL27 interactions with CCR10; however, the N-terminal Phe0 extends into a tiny flat subpocket near TM1, sandwiched between Q44(1.35) and L300(7.43). RTCNN calculations suggest that the resulting stacking of Phe0 with Q44(1.35) and L300(7.43) is highly favorable, even exceeding that of Phe1 (Fig. 3*I*). These contacts, absent in WT CCL27, likely explain the superagonist properties of F-CCL27. Consistent with experimental observation, *in silico* mutation of Phe0 into Leu0 produced less favorable packing and deteriorated RTCNN scores (Fig. 3*J*), likely because the aliphatic non-flat shape of Leu0, makes it unable to fit in the subpocket and engage in pi–stacking interactions.

### Fusing the N terminus of CCL28 onto the core domain of CCL27 enhances CCR10 activation

**Figure 4:**
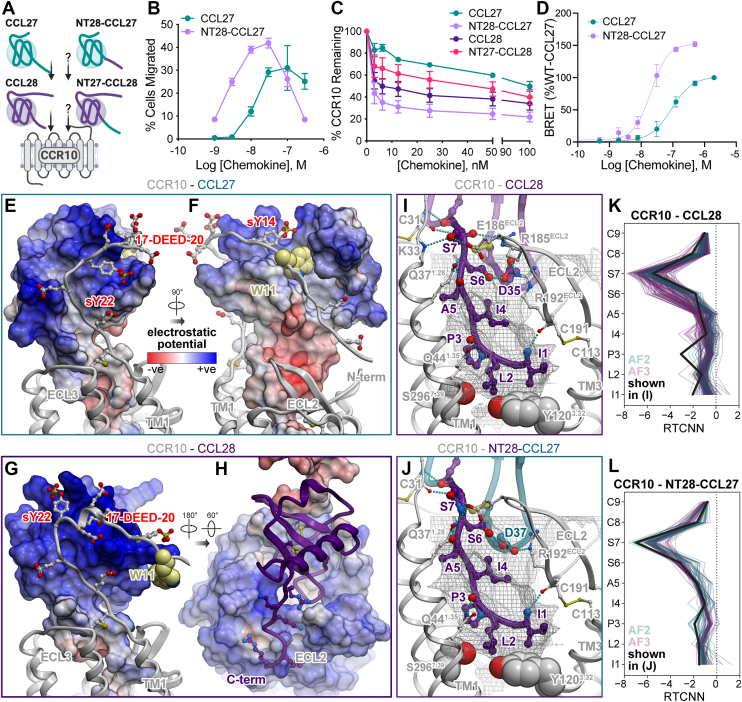
**Pharmacological and structural characterization of N terminally swapped chimeras of CCL27 and CCL28.***A*, *cartoon* depicting CCL27 and CCL28 specificity for CCR10 and unknown activity of N-terminal chimeras. *B*, cell migration analysis of WT CCL27 and NT28-CCL27 with L1.2/CCR10 cells across varying chemokine concentrations. Shown are representative data (mean ± SD) of independent experiments (n = 5) performed in technical duplicates or triplicates and plotted as the percent of cells migrated after a 2 h incubation at 37 °C. *C*, comparison of WT or mutant chemokine-mediated internalization of L1.2/CCR10 cells. Cells were treated with varying concentrations of chemokine, incubated at 37 °C for 45 min and then receptor levels were detected and plotted as the percent of receptor remaining compared to cells treated with no chemokine. Results shown are the mean ± SEM of single measurements obtained for each concentration combined from at least three independent experiments (n = 3–4). *D*, β-arrestin recruitment by BRET. Cells were transiently transfected with CCR10-RlucII and βarr2-GFP10 and then stimulated with varying concentrations of CCL27 or NT28-CCL27. The BRET ratios are shown as a % of WT-CCL27. Results shown are the mean ± SD of at least three independent experiments performed in technical duplicates or triplicates. *E* and *F*, interactions of the receptor N terminus with the globular core of the chemokine in the model of the CCR10–CCL27 complex. The complex is shown in two orientations; the chemokine is represented as a surface mesh colored by the electrostatic potential; the receptor is shown as a *ribbon* and *sticks*. Receptor residue W11 is shown in *light yellow spheres*. *G* and *H*, interactions of the receptor N terminus and extracellular loops with the globular core in the chemokine in the structural model of the CCR10–CCL28 complex. The complex is shown in two orientations. In (*G*), the chemokine is represented as a surface mesh colored by the electrostatic potential, while the receptor is shown as *ribbon* and *sticks*. In (*H*), the receptor is shown as the electrostatic potential mesh, while the chemokine is in *purple ribbon*. *I* and *J*, CRS2 interactions in the structural models of CCR10 (*white ribbon and spheres*) bound to CCL28 (*I*) and NT28-CCL27 (*J*). Chemokine segments originating from CCL28 are shown in *purple ribbon* and *sticks*; *teal ribbon* and *sticks* are segments from CCL27. The optimal ligand atom placement surface in the orthosteric pocket of the receptor is shown as *gray mesh*. The complexes are viewed along the plane of the membrane in the TM2-to-TM5 direction, TM2 is hidden for clarity. Residues Tyr120(3.32) and Ser296(7.39) of the receptor are shown in *gray spheres*. Hydrogen bonds are shown as *cyan dotted lines*. *K* and *L*, RTCNN scores of N-terminal residues of CCL28 (*K*) and NT28-CCL27 (*L*) across the ensembles of AF2 and AF3 structural models of CCR10 complexes with these two chemokine variants. AF, AlphaFold; BRET, bioluminescence resonance energy transfer; CCR, CC chemokine receptor; RTCNN, Radial and Topological Neural Network; TM, transmembrane.

To further understand the contributions of the N termini of CCL27 and CCL28 to receptor specificity and engagement, we generated N-terminal chimeras (Fig. 1, *E* and *F*) and examined their ability to activate CCR10 (Fig. 4*A*). NT28-CCL27, a chimera consisting of the N terminus of CCL28 and the globular core domain of CCL27, showed increased potency and efficacy in promoting the migration of L1.2/CCR10 cells compared to WT CCL27 (Fig. 4*B*), while NT27-CCL28 and F-NT27-CCL28 were equipotent to WT CCL28 (Fig. S4). CCL28 was also more potent than CCL27 in inducing internalization of CCR10 and the addition of the CCL28 N terminus onto CCL27 conferred NT28-CCL27 with a stronger capacity to internalize CCR10 than WT CCL27 (Fig. 4*C*). By contrast, NT27-CCL28 was less potent in internalizing CCR10 than WT CCL28, consistent with the more modest internalizing capacity of CCL27 (Fig. 4*C*). NT28-CCL27 also promoted β-arrestin recruitment to CCR10 with a 1.5-fold greater efficacy and 3.5-fold greater potency than WT CCL27 (Fig. 4*D*, Table S1). Thus, despite the relatively low sequence homology between the chemokine globular domains (∼30% identity), they still bind CCR10 in a manner that accommodates exchange of the chemokine N termini with preservation of some of the activation characteristics (*e.g.,* internalization) of the chemokine from which the N terminus was derived.

To rationalize the experimental data, we generated structural models of CCR10 complexes with CCL27 and CCL28 using AF2 and AF3 (Figs. 2, *D*, *E*, 4, *E–J*, S1 and S2, Supp. Data 1–3). The most striking differences between CCR10 recognition of CCL28 and CCL27 are observed in CRS1 (Fig. 4, *E*–*H*). With CCL28, the proximal N terminus of CCR10 including its acidic cluster 17-DEED-20 and the predicted sulfotyrosine sTyr22, folds into a short α-helix and favorably engages the chemokine’s basic 40s loop (46-KRR-48), whereas the distal N terminus of CCR10 is largely unutilized (Fig. 4*G*). By contrast, when bound to CCL27, the N terminus of CCR10 not only favorably interacts with the basic residues in its N-loop (Lys16), pi-helix (20-KLLRK-24), and 40s loop (R50) (Fig. 4*E*) but in some models also wraps around the chemokine (Fig. 4*F*). On the opposite side of CCL27, Trp11 in the receptor N terminus may pack favorably in the pocket formed by the β_1_-strand and the unique loop between the two C-terminal helices of CCL27 (Fig. 4*F*); this pocket does not exist in CCL28 because it lacks the second C-terminal helix (Fig. 1*C*). These observations suggest that the globular core of CCL27 provides a more favorable context for CCR10 CRS1 binding, compared to the globular core of CCL28.

In CRS2, the N terminus of CCL28 binds CCR10 in a manner similar to that of WT CCL27, with CCL28 Ile1 and Pro3 occupying approximately the same areas and cavities in the binding pocket as CCL27 Phe1 and Leu3, respectively (Figs. 2*D* and 4*I*). However, closer examination reveals differences in the conformation and interaction of the chemokines’ N termini. The binding geometry of CCL28 enable three additional hydrogen bonds with the CCR10 pocket residues: one between the N-terminal amine and the backbone oxygen on CCR10 C191(ECL2), another between the backbone oxygen of CCL28 Pro3 and CCR10 Q44(1.35), and the third between CCL28 Ser7 and the backbone of CCR10 K33(NT). Moreover, models predict an intramolecular hydrogen bond between CCL28 Ser6 and Asp35 in its 30s loop, which in turn bonds to CCR10 R185(ECL2) (Fig. 4*I*). In CCL27 complexes, analogous hydrogen bonds were not observed: for example, the residues in proximity of the corresponding Asp37 in the 30s loop of CCL27 are 4-PP-5, which lack hydrogen bond donor functionality (Fig. 2*D*). Altogether, these gained interactions contribute to a more favorable interaction profiles, per RTCNN, for the N termini of CCR10-CCL28 complexes (Fig. 4*K*) compared to CCR10-CCL27 (Fig. 2*F*), with an especially large “dip” (favorable scores) at Ser7 and (in many AF3 models) at Ser6 of CCL28 (Fig. 4*K*).

When the N terminus of CCL28 is grafted onto the globular core of CCL27 (NT28-CCL27), the result combines the best of both complexes including the favorable CRS1 interactions of the CCL27 globular core (Fig. 4, *E* and *F*), CRS2 interactions of the CCL28 N terminus (Fig. 4, *I*–*L*), and the hydrogen bond between the proximal chemokine N terminus and the 30s loop (Fig. 4*J*). This possibly explains the improved agonist properties of this chimera compared to both WT CCL27 and CCL28 (Fig. 4, *B*–*D*). By contrast, grafting the N terminus of CCL27 onto the globular core of CCL28 (NT27-CCL28) does not produce such synergy, explaining the lack of enhanced agonist potency (Fig. 4*C* and S4).

### Swapping the N termini of CCL27 and CCL28 does not lead to CCR3 activation

**Figure 5:**
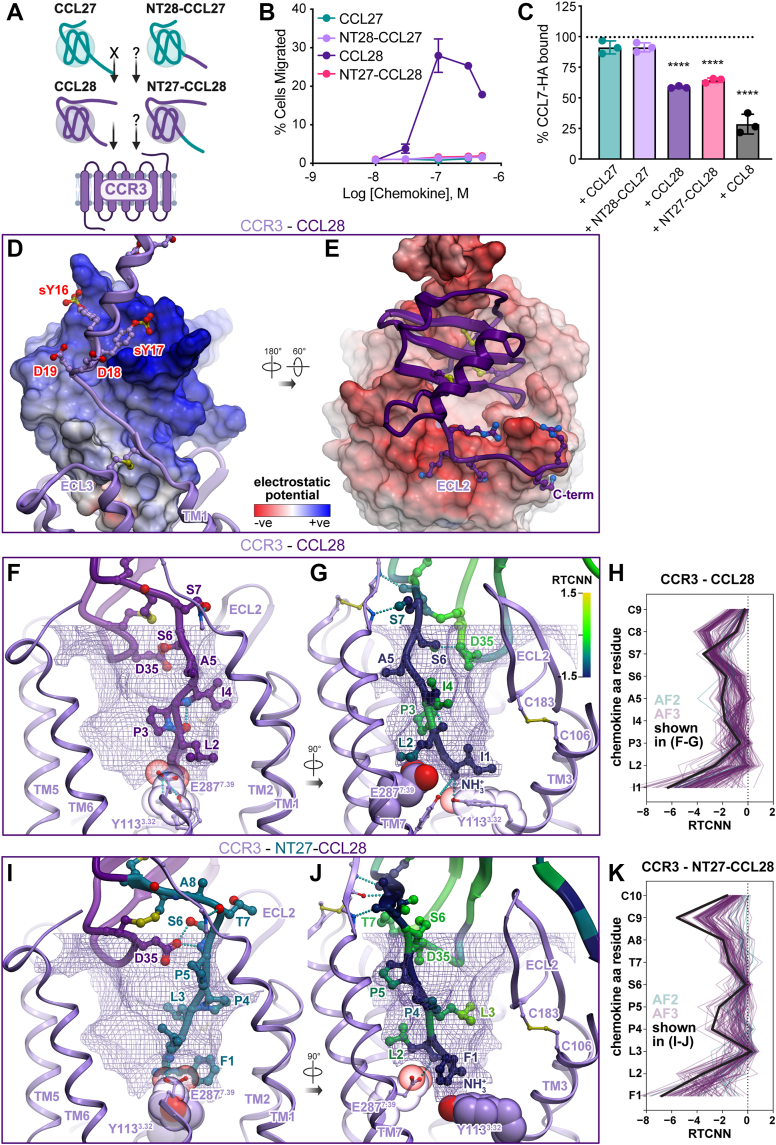
**Functional and structural analysis of N-terminal chimeras of CCL27 and CCL28 with CCR3.***A*, *cartoon* depicting CCL28 specificity for CCR3, lack of activity by CCL27, and unknown activity of N-terminal chimeras. *B*, cell migration analysis of WT CCL27, WT CCL28, and N-terminal chimera mutants (NT28-CCL27 and NT27-CCL28) with L1.2/CCR3 cells across varying chemokine concentrations. Shown are representative data (mean ± SD) of independent experiments (n = 3) performed in technical duplicates, plotted as the percent of cells migrated after 2 h incubation at 37 °C. *C*, competition binding experiments were performed on L1.2/CCR3 cells using C terminally HA-tagged CCL7 (CCL7-HA). Cells were incubated with 250 nM of CCL7-HA alone or in combination with a 4-fold excess of competing ligand (1000 nM) for 30 min on ice, cells were washed and then CCL7 levels were determined by flow cytometry using an anti-HA antibody. Results shown are the mean ± SEM of single measurements obtained for each concentration combined from independent experiments (n = 3); the *dotted line* depicts 100% retention of bound CCL7-HA. Statistical analysis was performed by ordinary one-way ANOVA with *post hoc* Dunnett’s multiple comparisons test. (∗∗∗∗*p* < 0.0001; CCL7-HA + CCL27, *p* = 0.1026; CCL7-HA + NT28-CCL27, *p* = 0.1026). *D* and *E*, the interactions between the globular domain of CCL28 and the N terminus (*D*) or extracellular loops (*E*) of CCR3. The complex is shown in two orientations. In (*D*), chemokine is represented as a surface mesh colored by the electrostatic potential, while the receptor is shown as *ribbon* and *sticks*. In (*E*), the receptor is shown as the electrostatic potential mesh while the chemokine is in *purple ribbon*. *F-G* and *I*-*J*, CRS2 interactions in the structural models of CCR3 (*violet ribbon and spheres*) bound to CCL28 (*F*-*G*) or NT27-CCL28 (*I*-*J*). The optimal ligand atom placement surface is shown as *violet mesh*; CCR3 residues Tyr113(3.32) and Glu287(7.39) as *spheres*. In (*F*) and (*I*), complexes are viewed parallel to the plane of the membrane in the TM7-to-TM3 direction; TM7 is partially hidden for clarity; chemokine segments originated from CCL28 are shown in *purple sticks and ribbons*; those from CCL27 are *teal sticks and ribbons*. In (*G*) and (*J*), complexes are viewed parallel to the plane of the membrane in the TM2-to-TM5 direction; TM2 is partially hidden for clarity; color represents per-atom RTCNN scores aggregated to residue backbones (for *ribbons* and *backbone sticks*) and side chains (for side chain sticks). *H* and *K*, RTCNN scores of N-terminal residues of CCL28 (*H*) and NT27-CCL28 (*K*) across the ensembles of AF2 and AF3 structural models of CCR3 complexes with these two chemokine variants. AF, AlphaFold; CCR, CC chemokine receptor; RTCNN, Radial and Topological Neural Network; TM, transmembrane.

In addition to CCR10, CCL28 activates CCR3, while CCL27 is a CCR10-selective ligand (Fig. 5*A*). In order to determine the requirements for CCR3 binding and activation and the specificity of the two chemokines, N-terminal chimeras of CCL27 and CCL28 were compared for their ability to promote migration of CCR3-expressing L1.2 cells in a bare filter chemotaxis assay. As shown in Figure 5*B*, NT28-CCL27 was unable to promote migration or compete with another CCR3 ligand (CCL7-HA) for binding the receptor (Fig. 5*C*), suggesting this chimera has little or no affinity for CCR3. The NT27-CCL28 chimera was also incapable of activating CCR3 (Fig. 5*B*) even though it still competed with CCR3 agonist CCL7 for receptor binding (Fig. 5*C*). This suggests that the N terminus and the globular domain of CCL28 work together to determine receptor selectivity and agonist activity, and that the N-terminal domain of CCL27 is incompatible with CCR3.

**Figure 6:**
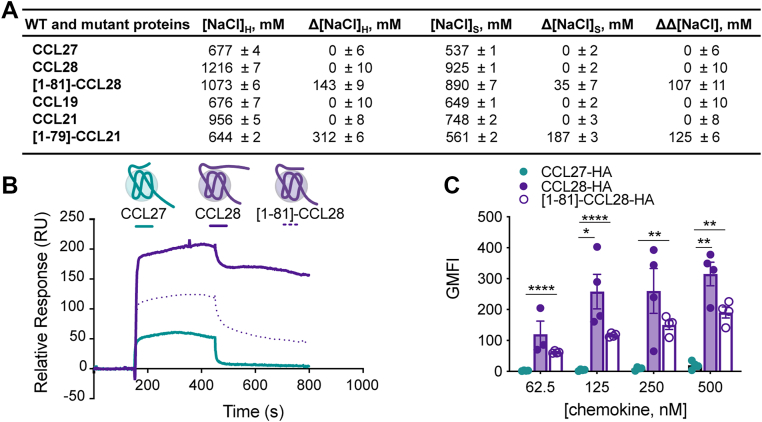
**GAG binding characterization of WT and mutant proteins.***A*, GAG binding characterization of CCL27, CCL28, CCL19, CCL21, and mutants by heparin sepharose chromatography. The concentration of NaCl (mM) required to elute protein from a heparin or a SP sepharose affinity column is reported as [NaCl]_H_ and [NaCl]_S_, respectively. Δ[NaCl] values represent the difference in [NaCl] required to elute mutant *versus* WT protein (Δ[NaCl] = [NaCl]^WT^ − [NaCl]^mutant^) from either heparin sepharose (Δ[NaCl]_H_) or SP-sepharose (Δ[NaCl]_S_). ΔΔ[NaCl] is a measure of the specificity of interaction, where ΔΔ[NaCl] = Δ[NaCl]_H_ –Δ[NaCl]_S_. A positive specificity index for a mutant means that the difference between the amount of NaCl required to elute the mutant from the heparin-sepharose column as compared to WT was greater than the same difference for the SP-sepharose column, suggesting specificity for heparin beyond electrostatic interactions generally associated with affinity for SP-sepharose. All data represent the mean ± SD of three independent experiments. *B*, SPR sensorgrams of CCL27, CCL28, and [1-81]-CCL28 showing the resulting signals (relative response in RU) for interaction with immobilized HS with the same concentration of injected chemokine (1000 nM concentration). *C*, CCL27, CCL28 and [1-81]-CCL28 binding of CHO-K1 cells expressing endogenous HS. C terminally HA-tagged CCL27, CCL28, or [1-81]-CCL28 was used to determine the extent of chemokine binding to CHO-K1 cells. Cells were incubated with varying concentrations of HA-tagged chemokine for 30 min on ice, washed and the geometric mean fluorescence intensity (GMFI) detected by flow cytometry using an anti-HA antibody normalized to an isotype control. Data shown is the mean ± SEM from single measurements obtained for each concentration combined from independent experiments (n = 3–4). Statistical analysis was performed by mixed-effects analysis with *post hoc* Tukey’s multiple comparisons test. (∗∗∗∗*p* < 0.0001; ∗∗*p* < 0.01; ∗*p* < 0.05; CCL27-HA: CCL28-HA at 62.5 nM, *p* = 0.1915; CCL28-HA: [1-81]-CCL28-HA at 62.5 nM, *p* = 0.4916; CCL28-HA: [1-81]-CCL28-HA at 125 nM, *p* = 0.1659; CCL27-HA: CCL28-HA at 250 nM, *p* = 0.0798; CCL28-HA: [1-81]-CCL28-HA at 250 nM, *p* = 0.4024; CCL28-HA: [1-81]-CCL28-HA at 500 nM, *p* = 0.0812). BRET, bioluminescence resonance energy transfer; CCR, CC chemokine receptor; GAG, glycosaminoglycan; HS, heparan sulfate; SPR, surface plasmon resonance.

To further dissect the molecular details underlying chemokine selectivity for CCR3 *versus* CCR10, we examined the amino acid composition of their orthosteric binding pockets (Fig. S5). Compared to the more canonical binding pocket composition of CCR3 (many bulky residues conserved among most chemokine receptors), the binding pocket of CCR10 features multiple large-to-small residue substitutions. For example, CCR3 residues W90(2.60), H114(3.33), and Y172(4.65) correspond to Ala98(2.60), Ser121(3.33), and Ser180(4.65) in CCR10 (Fig. 6). Additionally, the ECL2 of CCR3 is strongly acidic, featuring several negatively charged residues (*e.g.*, Glu173, Glu175, Glu176, Glu189), whereas the ECL2 of CCR10 is more neutral/basic (*e.g.*, Gln181, Gln184, Arg185, Arg192, Glu197, Gln201) (Figs. 4*H*, 5, *D*, *E* and S5*A*).

The AF models of the CCR3-CCL28 complex (Figs. S1 and S2, Supp. Data 1–3) suggest that both of these factors contribute to the high affinity and potency of CCL28 (but not CCL27) toward CCR3 (Fig. 5, *D*–*G*). Despite reduced size, compared to CCR10 (Fig. S5, *B* and *C*), the CCR3 binding pocket favorably accommodates the smaller CCL28 N terminus (Fig. 5, *F*–*H*) in the geometry loosely resembling its complex with CCR10 (Fig. 4*I*). The side chain of Ile1 packs underneath the ECL2 of CCR3 and the conserved disulfide bridge Cys106(3.25)-Cys183(ECL2) (Fig. 5, *G* and *H*). Leu2 of CCL28 does not reach Y113(3.32) in the CCR3 complex (Fig. 5*G*) as it does in the CCR10 complex (Fig. 4*I*). Instead, it is above the residue at position 7.39, which in CCR3 is a Glu (Glu287(7.39)), as in most other chemokine receptors; it is Ser296(7.39) in CCR10 (Fig. S5). The N-terminal amine of CCL28 hydrogen-bonds to the hydroxyl groups of Y113(3.32) and Y291(7.43) (Fig. 5*G*), likely stabilizing the active conformation of the TM bundle. In CRS1, the acidic residues in the proximal N terminus of CCR3 favorably interact with the basic patches in the CCL28 4_10_ helix and 40s loop (Fig. 5, *D* and *E*). A major contribution to the binding energy seems to be the charge–charge interaction between the very basic β_1_-strand and C terminus of the chemokine on one side, and the acidic ECL2 of the receptor on the other (Fig. 5*E*).

We also generated a series of models of CCR3 with NT27-CCL28 (Figs. 5, *I*–*K*, S1 and S2, Supp. Data 1–3) to illustrate why this chimeric chemokine continues to bind but cannot activate the receptor. The model suggests that the longer N terminus of CCL27, which also has a bulkier Phe residue in position 1, cannot fit in the smaller binding pocket of CCR3 in the orientation that in other complexes provides activation-associated contacts. Despite this, the CRS1, ECL2 (Fig. 5, *D* and *E*), and CRS2 (Fig. 5, *I*–*K*) interactions with this chimera continue to be favorable and likely drive the binding observed experimentally (Fig. 5*C*).

### CCL28 binds GAGs with significantly higher affinity than CCL27, providing another indication of the nonredundant functions of these chemokines

In addition to binding chemokine receptors, chemokines interact with the GAG chains of proteoglycans on cell surfaces and the extracellular matrix as a mechanism to localize them in chemokine gradients that provide directional cues for migrating cells (34, 61, 62). We previously demonstrated that CCL27 interacts weakly with GAGs and has a diffuse GAG binding surface (28), while characterization of CCL28 binding to GAGs has been limited (63). To assess the GAG-binding propensities of CCL28, we employed heparin-sepharose (HP) affinity chromatography, which is commonly used to approximate the relative affinities of heparin-binding proteins based on the concentration of NaCl required to elute the proteins from an HP column (64) (Fig. 6*A*). For CCL28, ∼1.2 M NaCl was required for elution, suggesting that it is a potent heparin-binding protein, in stark contrast to CCL27 that showed a much weaker interaction, requiring only ∼680 mM NaCl for elution (Fig. 6*A*). We also evaluated whether the extended C terminus of CCL28 contributes to GAG binding, similar to CCL21 and CXCL12γ (65, 66). To test this, we generated [1-81]-CCL28, which lacks most of the 28-residue extension, but maintains the unusual third disulfide that links the β1-strand of the globular domain to a region just past the C-terminal α-helix (Fig. 1*B*). The [1-81]-CCL28 truncation mutant eluted at ∼1.1 M NaCl, 143 mM less than that required for WT CCL28 (Fig. 6*A*), indicating that while the C terminus contributes to GAG binding, the globular domain contributes to the bulk of the GAG-binding affinity consistent with its highly basic surface (Fig. S6). For comparison, we evaluated two other well-characterized chemokines, CCL19, CCL21, and a C-terminal truncation mutant of CCL21, [1-79]-CCL21 (Fig. 6*A*). CCL21 is known to bind GAGs with high affinity and in our hands eluted from the HP column at a NaCl concentration of 956 mM, whereas CCL19 is considered to be a more diffusible, weak-binding chemokine, and eluted at 676 mM NaCl, consistent with prior studies (63, 65, 67, 68). Moreover, removal of the C-terminal tail of CCL21 resulted in a variant with a heparin-binding propensity similar to CCL19, as demonstrated previously (68). Thus, while CCL27 is similar to CCL19 in its GAG binding affinity, CCL28 has an even higher affinity than CCL21; and even after deletion of the CCL28 C-terminal tail, it still retained an affinity equivalent to WT CCL21 ([NaCl]_H_ ∼ 1 M) (Fig. 6*A*).

Affinity values and association/dissociation rates from surface plasmon resonance (SPR) experiments supported the above conclusions (Figs. 6*B* and S7). For the SPR experiments, biotinylated heparan sulfate (HS) was immobilized on neutravidin-coated BIAcore C1 chips and varying concentrations of WT or mutant chemokine flowed over the chip, as previously described (69, 70) (Fig. S8). In qualitative agreement with the HP affinity chromatography data, the SPR results revealed an ∼1000-fold difference in the affinity of CCL27 and CCL28 for HS (3.2 μM *versus* 3 nM, respectively, (Figs. 6*B* and S7). The C-terminal truncation mutant, [1-81]-CCL28, exhibited only slightly weaker affinity for HS relative to WT CCL28 (15 nM *versus* 3 nM) in contrast to the complete loss of binding observed for the CCL21 C-terminal truncation mutant, [1-79]-CCL21 (Figs. 6*B* and S7).

To determine whether the heparin-binding propensities of CCL27 and CCL28 correlate with their ability to bind cell surface GAGs, we incubated C terminally hemagglutinin (HA)-tagged CCL27, CCL28 or [1-81]-CCL28 with CHO-K1 cells, which endogenously express HS, and measured bound chemokine by flow cytometry using an anti-HA antibody (Fig. 6*C*). These data revealed substantial accumulation of CCL28 and diminished but still appreciable interaction of [1-81]-CCL28, consistent with the HP sepharose and SPR data (Figs. 6, *A*, *B* and S7). Together, these data highlight additional nonredundant properties of two chemokines that share CCR10 as their receptor.

## Discussion

CCL27 and CCL28 are both involved in epithelial immunity through their common receptor CCR10, but in different capacities: CCL27 is primarily associated with skin where it attracts T cells, while CCL28 is expressed by mucosal tissue and recruits antibody-producing B lymphocytes (8, 71, 72). Given their clear functional differences, prior studies have sought to determine if the ligands differ in their signaling bias, that is, their ability to preferentially activate G protein *versus* β-arrestin pathways downstream of CCR10 (73, 74). However, there has been little in the way of structure-function studies, which can provide insight into activation mechanisms and lead to the discovery of variants with altered pharmacological properties.

In this study, we investigated how CCL27 and CCL28 activate CCR10 with a combination of N-terminal mutations, deletions, and chimeric swaps of the chemokines, coupled with AF modeling of chemokine complexes with CCR10. Key findings include the discovery that the first two amino acids (Phe1 and Leu2) of CCL27 are critical for its ability to activate CCR10. AF modeling and RTCNN scoring suggest that these two residues trigger CCR10 activation upon interaction with residues at positions 3.32 and 7.39 in the receptor binding pocket that are known to be involved in the activation of homologous receptors (49, 50, 51, 52, 53). Notably, the two-residue deletion mutant resulted in an antagonist variant ([3-88]-CCL27) that may provide the starting point for the rational design of higher affinity antagonists that could have therapeutic value. For example, CCL27 has previously been implicated in atopic dermatitis due to its ability to recruit CCR10 expressing CD4+ and CD8+ T cells; moreover, administration of an anti-CCL27 neutralizing antibody reduced dermal inflammation along with T and mast cell accumulation, T cell proliferation and the presence of inflammatory cytokines (15). CCR10 has also been implicated in rheumatoid arthritis although the efficacy of inhibiting the receptor on disease pathogenesis remains to be determined (75). Numerous reports have described a role of CCR10 and its ligands in cancer although the benefits of CCR10 antagonism may be cancer-dependent: the receptor can have protumoral effects when expressed on regulatory T cells or tumor cells or antitumoral effects when expressed on CD4+ and CD8+ T cells (76, 77, 78, 79). Thus, [3-88]-CCL27, or higher affinity analogues, may be useful for further probing the efficacy of inhibiting CCR10 for the treatment of these diseases and potentially used directly as protein therapeutics.

We also showed that N-terminal extension of CCL27 with a Phe results in a superagonist that is substantially more potent (10-fold) and efficacious (1.1-fold) than WT CCL27 in promoting cell migration in a bare filter assay, with even more enhanced effects observed in inducing transendothelial cell migration and β-arrestin recruitment. AF models of the CCR10–F-CCL27 complex, and the associated residue RTCNN scores, suggest this is due to the extension of Phe0 into a cryptic subpocket proximal to TM helices 1 and 7, unique to CCR10 *versus* most other chemokine receptors. The Phe addition may result in greater stabilization of the active receptor conformation and/or increased residence time of the ligand on the receptor; however, the CCR10–CCL27 system proved recalcitrant to traditional radioligand binding assays, and therefore affinities and off-rates were not determined. Regardless of mechanism, F-CCL27 may be useful as an adjuvant to enhance the efficacy of vaccines: for example, plasmids encoding CCL27 along with the SARS-CoV-2 spike protein resulted in increased anti-spike protein antibodies to CD8+ T cells at mucosal surfaces, which conferred significantly enhanced immunity against SARS-CoV-2 (80). Both CCL27 and CCL28 have also been shown to provide immunity against influenza and HIV (30, 31, 81) due to the fact that adjuvant activity is dependent on CCR10-expressing plasma B cells that produce IgA and accumulate at mucosal surfaces (14). However, whether superagonism conferred by F-CCL27 results in a more potent response *in vivo* remains to be determined.

In contrast to CCL27, extension of the N terminus of CCL28 with a Phe (or Phe-Phe) did not enhance its ability to promote CCR10-mediated cell migration. These contrasting results illustrate that superagonism through N-terminal extension is not a universal or generalizable phenomenon even for a single receptor, CCR10, let alone for the entire chemokine receptor family. The N-terminal lengths and binding geometries for the chemokines in various receptor-chemokine pairs vary dramatically (37, 82). The contributions of the chemokine N-terminal domain to binding and agonism also vary (56, 83) and in fact extensions can produce superagonists for some chemokines, but antagonists or even completely abrogate binding for others (39, 41, 42, 43, 44).

Although CCL28 extension did not improve its agonist properties, the impact of swapping the N termini of CCL28 and CCL27 on CCR10-mediated migration and internalization suggests that the CCL28 N terminus is a stronger driver of agonist responses. And in fact, the chimera (NT28-CCL27) also proved to be a superagonist of CCR10 that like F-CCL27 may have therapeutic utility. In addition to having potential as more potent vaccine adjuvants than the WT ligands, superagonists may also serve as "functional antagonists" if they enhance receptor internalization, as we observe for F-CCL27 and NT28-CCL27, especially if internalization results in trafficking to degradation pathways and/or inhibition of receptor recycling (84, 85, 86). Prior examples of chemokine-based functional antagonists include AOP-RANTES/CCL5 and PSC-RANTES/CCL5, both of which inhibit HIV entry into cells by promoting internalization and intracellular retention of CCR5 (58, 87).

In addition to identifying variants of CCL27 with distinct pharmacological properties, AF models of complexes of CCR10 and CCR3 with CCL27 and CCL28 also provided insight into the ability of CCR10 to be activated by both CCL27 and CCL28 and the specificity of CCR3 for CCL28. Specifically, the orthosteric pocket of CCR3 is too small for the longer/bulkier N terminus of CCL27 but accommodates that of CCL28. CCL28 (pI = 10.35) is also more basic than CCL27 (pI = 9.1) (Fig. S6) and shows electrostatic complementarity with the acidic N terminus and ECL2 of CCR3, which is lacking in CCL27. The more basic nature of CCL28 translates into its higher affinity for heparin, HS, and cell surface GAGs than CCL27, which may be critical for the accumulation of CCL28 in mucosal tissues in the context of its chemoattractant functions. For example, HS within the mouse caecum (colon) is expressed at low levels in naïve tissue and upregulated at the base of the crypts following chronic infection (88), which may benefit from the intrinsic high-affinity (and slow off-rate) HS-binding properties of CCL28 for directed immune cell migration to focused regions of HS expression. On the other hand, expression of HS GAGs on the skin is localized to the vasculature, immune cell surface, and epidermis at rest and following inflammation (89), perhaps providing sufficient sites of retention for the more weakly HS-binding CCL27. Similar to defensins and histatins (90, 91), the abundance of positively charged residues is also important for the broad-spectrum antimicrobial activity of CCL28, which facilitates disruption of bacterial membranes (90). Thus, CCL28 has been described as having a dual role in mucosal protection through its ability to recruit CCR3-and CCR10-expressing cells and its antimicrobial activity (91).

Altogether, the data in this study provide mechanistic explanations for the nonredundant biological functions of CCL27 and CCL28 along with structural insights that account for their receptor selectivity and distinct context-dependent signaling. The pharmacologically unique antagonist and superagonist variants discovered in the course of our studies may prove useful for interrogating the value of targeting CCR10 for specific diseases.

## Experimental procedures

### Chemokine expression and purification

WT and mutant CCL27 and CCL28 and CCL7-HA were expressed as His-ubiquitin fusions using the pHUE vector and purified from inclusion bodies according to established procedures (28, 69). CCL8 was expressed and purified with a N-terminal His(8×)-tag followed by an enterokinase cleavage site, similar to previously established procedures (92). Protein identity and purity was confirmed by electrospray ionization mass spectrometry (Scripps Center for Metabolomics or the UC San Diego Molecular Mass Spectrometry Facility). Specified mutants were generated by QuikChange site-directed mutagenesis (Stratagene) and purified in the same manner as the WT protein.

### Cell culture

Murine L1.2 pre-B cells stably expressing human CCR10 (kind gift of Eugene Baker, Stanford University) or human CCR3 (generated in-house through limiting dilution) were maintained in RPMI 1640 media (Gibco) supplemented with 10% fetal bovine serum (FBS) (Gibco), 1% minimal essential medium nonessential amino acids, 1% sodium pyruvate, 0.1% β-mercaptoethanol, and 300 μg/ml geneticin and grown at 37 °C with 5% CO_2_. EA926 human umbilical vein endothelial cells (kind gift of the Shyy lab, UCSD) were cultured in Dulbecco's modified Eagle's medium/10% FBS and grown at 37 °C with 5% CO_2_. HEK293 cells (American Type Culture Collection, cat# CRL-1573) were maintained in Dulbecco's modified Eagle's medium/10% FBS and grown at 37 °C with 5% CO2.

*Mycoplasma* testing was performed using either the MycoStrip (InvivoGen) or MycoAlert Plus (VWR) *mycoplasma* detection kit, with no *mycoplasma* detected.

### DNA plasmids and cloning

CCR10-RLucII was generated by subcloning the CCR10 coding sequence into a vector containing the RLucII tag. The βarr2-GFP10 construct was kindly gifted by Nikolaus Heveker (Université de Montréal).

### Chemokine binding on CHO cells

Binding of C terminally HA-tagged chemokine on CHO-K1 cells (kind gift of Dr Jeffrey Esko, UC San Diego) was performed essentially as described previously (69) with a few minor modifications. Cells were treated with HA-tagged WT or mutant chemokine (62.5–500 nM) prepared in flow cytometry buffer (PBS + 0.5% bovine serum albumin) and incubated on ice for 30 min. Unbound chemokine was removed by washing cells three times with flow cytometry buffer. Chemokine levels were then detected by incubation with a phycoerythrin (PE)-conjugated anti-HA monoclonal mouse antibody (Miltenyi Biotec, cat# 130-092-257) or PE-conjugated mouse isotype control (BD, cat #555787), according to manufacturer instructions and analyzed by a Guava EasyCyte 8HT flow cytometer (EMD Millipore). Postacquisition analysis with FlowJo (https://www.flowjo.com/flowjo/overview; Tree Star, Inc) was used to determine the geometric mean fluorescent intensity for each sample normalized to isotype control; data are plotted as the mean ± SD of at least three independent experiments.

### Bare filter and transendothelial cell migration assays

Traditional bare filter migration assays were performed as described previously (69, 70) with some modifications. Briefly, cells were resuspended at 1.5 to 2.5 × 10^6^ cells/ml in RPMI 1640 + 10% FBS and 100 μl of cells were added to the upper chamber of a 5 μm pore size 24-well transwell filter insert (Corning), or 75 μl of cells added to the upper chamber of a 96-well transwell setup (Corning). Cells were allowed to migrate toward varying concentrations of CCL27 or CCL28 present in the lower chamber. After 2 h of incubation at 37 °C/5% CO_2_, migrated cells were counted on a Guava EasyCyte 8HT flow cytometer (EMD Millipore) by counting the number of cells present in 30 s. Migration was calculated as the percent of migration compared to the maximal number of possible cells migrated (no filter). To determine whether chemokine variants retained their ability to bind to surface receptor, competition binding migration assays were also employed. In these instances, bare filter migration assays were performed similar to above; however, each well contained 50 nM WT CCL27 in addition to increasing concentrations of mutant CCL27. While these assays do not allow the quantification of binding constants, they reveal whether a chemokine mutant retains the ability to interact with a receptor in a manner competitive with WT chemokine. Transendothelial migration assays were performed in a manner identical to the bare filter assays, with the exception that filters were first coated with 0.1 mg/ml type I collagen (Purecol, Advanced Biomatrix) for 1 h at 37 °C and then EA926 cells were added to each well (0.1 × 10^6^ cells/filter in 100 μl) and allowed to grow to confluency for 2 days at 37 °C with 5% CO_2_. After 2 days, transendothelial migration assays were performed as described for the bare filter experiments. Assays were undertaken in duplicate or triplicate with at least three independent experiments performed for each dataset. Representative data are plotted as the mean ± SD, unless otherwise stated.

### Chemokine competition binding assays

Competition binding experiments were performed on L1.2/CCR3 cells using C terminally HA-tagged CCL7 (CCL7-HA). CCR3 expression was induced by incubation of cells with 5 mM sodium butyrate for ∼22 h prior to conducting the experiment. Cells were then incubated with 250 nM of CCL7-HA alone or in combination with a 4-fold excess of competing ligand (1000 nM) for 30 min on ice, cells were washed and then CCL7-HA levels were determined by flow cytometry using an anti-HA antibody. Results shown are the mean ± SD of single measurements obtained for each concentration combined from three independent experiments.

### Heparin-sepharose binding assays

The amount of NaCl required to elute CCL27, CCL28, CCL19, CCL21, and mutants from a 1-ml HiTrap Heparin HP column (GE Healthcare) or a 1-ml HiTrap SP HP column (GE Healthcare) was determined as previously described (70). Each assay was performed in triplicate; data are plotted as the mean ± SD.

### Surface plasmon resonance

A BIAcore 3000 instrument (GE Healthcare) was used to perform SPR using a C1 SPR chip coated with HS, as described previously (69, 70). Briefly, paired cells on a C1 SPR chip (GE Healthcare) were equilibrated in running buffer (10 mM Hepes, 150 mM NaCl, 3 mM EDTA, 0.05% Tween-20, pH 7.4) before activation with NHS:EDC (1:1 mixture) and immobilization of neutravidin (0.2 mg/ml), until saturation, in 20 mM sodium acetate, pH 6.0. Excess neutravidin was removed by washing with regeneration buffer (0.1 M glycine, 1 M NaCl, 0.1% Tween-20, pH 9.5). Remaining active sites were blocked by flowing 1 M ethanolamine over the paired cells. Biotinylated HS (0.2 mg/ml) (69) was then flowed over one of the paired flow cells followed by extensive washing with running and regeneration buffer. Chemokines, at the indicated concentrations, were then passed over the paired cells in running buffer (40 μl/min, to limit mass transfer effects) and the HS specific response monitored by subtracting the signal from the paired flow cell without HS from that with HS. The surface was regenerated between chemokine injections using regeneration buffer. The resulting sensorgrams were then analyzed using BIAevaluation software and apparent dissociation constants (*K*_D_) were obtained using a 1:1 Langmuir interaction model and/or steady-state analyses where possible (69, 93). Fitting of the data were assessed visually and using the Chi^2^ values, where a Chi^2^ <10 was considered to be indicative of a good fit. In instances where the HS-bound chemokine signal reached saturation during injection at a sufficient range of concentrations, these maximum signal values were plotted against concentration and analyzed with the 1:1 steady-state affinity model equilibrium analysis in the BIAevaluation software (GE Healthcare). The resulting affinities are considered to be “apparent affinities” due to issues associated with analyzing chemokine:GAG interactions discussed in detail elsewhere (64, 69).

### Calcium flux assays

Chemokine-mediated calcium flux was measured using the Screen Quest Fura-2 No Wash Calcium Assay Kit (AAT Bioquest). Briefly, 100 μl of 2 × 10^5^ L1.2/CCR10 cells suspended in 1X Hank’s balanced salt solution with 20 mM Hepes (HHBS, pH 7.2) were seeded in a black-wall, clear-bottom assay plates. The plates were centrifuged at 140*g* for 5 min at 25 °C and incubated for 30 min at 37 °C with 5% CO_2_. Then, 100 μl of Fura-2 AM dye-loading solution prepared in HHBS was added to the cells, followed by 1 h incubation at 37 °C with 5% CO_2_. After that, the plate was loaded to the TECAN Spark multimode microplate reader for receptor desensitization measurements. A TECAN Spark multimode microplate reader was used for all measurements with assays performed at 37 °C. For desensitization experiments, chemokine (10 μl in HHBS with 1% bovine serum albumin) were injected into wells using the Te-Inject module after 10th (first injection) and 75th (second injection) reading cycles. Fluorescence intensity measurements were made with excitation at 340/380 nm and emission at 510 nm. Relative fluorescence units were calculated by the ratio of 510 nm emission to the 340 and 380 nm excitation intensities and normalized to baselines values from the first 10 cycles. The processed data were plotted using GraphPad Prism (GraphPad Software; https://www.graphpad.com/features).

### Receptor internalization assays

Internalization assays were performed as described previously (94), with some modifications. Briefly, L1.2 cells expressing CCR10 were prepared at 1 × 10^6^ cells/ml in assay buffer (RPMI + 10% FBS) and chilled on ice in a 96-well plate. Cells were spun down to remove assay buffer and resuspended in 100 μl (100,000 cells/sample) of varying concentrations (3.1–100 nM) of WT or mutant chemokine prepared in cold assay buffer followed by incubation at 37 °C/5% CO_2_ for 45 min. Cells were then diluted in cold flow cytometry buffer and washed twice with flow cytometry buffer. CCR10 surface levels were measured by staining cells with an anti-hCCR10 PE-conjugated rat mAb (R&D, cat# FAB3478P) or isotype control according to manufacturer instructions. Data were acquired on a Guava EasyCyte 8HT flow cytometer (EMD Millipore) and analyzed with FlowJo software (Treestar, Inc.). Data were plotted using GraphPad Prism (GraphPad Software) and shows the percent of CCR10 remaining on the cell surface (compared to the maximal CCR10 levels detected with no chemokine added) from three independent experiments (mean ± SD).

### BRET assays

For evaluation of β-arrestin recruitment to CCR10, HEK293 cells were transfected with Mirus TransIT-Lt1 reagent when cells reached ∼70% confluency. Transfection included 100 ng of BRET donor (CCR10-RlucII) and 2 μg of BRET acceptor (βarr2-GFP10). Assays were performed ∼48 h posttransfection. On the day of the assay, cells were seeded into 96-well plates (100,000 cells per well) and incubated in Tyrode's Buffer (140 mM NaCl, 12 mM NaHCO_3_, 5.6 mM D-Glucose, 2.7 mM KCl, 1 mM CaCl_2_, 0.5 mM MgCl_2_, 0.37 NaH_2_PO_4_, 25 mM Hepes) for 40 min followed by the addition of the substrate, Prolume Purple (NanoLight Technologies) to a final concentration of 5 μM. After 10 min, baseline BRET measurements were performed to measure donor and acceptor emissions at their respective wavelengths: 480 nm and 530 nm. Subsequently, the indicated concentrations of chemokines were added to wells and BRET measurements were taken every 5 min for 30 min. The average time of each reading was calculated, and raw BRET values were converted into a BRET ratio (donor/acceptor). Data were plotted using GraphPad Prism (GraphPad Software) and represented as the inverse of the BRET ratio and normalized to the maximal WT response. All data points are the mean ± SD from a minimum of three independent experiments performed in at least duplicates.

### Molecular modeling

Structural models of CCR3 and CCR10 complexes with WT and mutant chemokines were constructed by AF2 Multimer v2.3.2 (95, 96) and AF3 (97), both locally installed on the UCSD Triton Shared Computing Cluster. The following amino acid sequences were used for modeling.

CCR10(2-331):

GTEATEQVSWGHYSGDEEDAYSAEPLPELCYKADVQAFSRAFQPSVSLTVAALGLAGNGLVLATHLAARRAARSPTSAHLLQLALADLLLALTLPFAAAGALQGWSLGSATCRTISGLYSASFHAGFLFLACISADRYVAIARALPAGPRPSTPGRAHLVSVIVWLLSLLLALPALLFSQDGQREGQRRCRLIFPEGLTQTVKGASAVAQVALGFALPLGVMVACYALLGRTLLAARGPERRRALRVVVALVAAFVVLQLPYSLALLLDTADLLAARERSCPASKRKDVALLVTSGLALARCGLNPVLYAFLGLRFRQDLRRLLRGGSCP

CCR3(2-322):

TTSLDTVETFGTTSYYDDVGLLCEKADTRALMAQFVPPLYSLVFTVGLLGNVVVVMILIKYRRLRIMTNIYLLNLAISDLLFLVTLPFWIHYVRGHNWVFGHGMCKLLSGFYHTGLYSEIFFIILLTIDRYLAIVHAVFALRARTVTFGVITSIVTWGLAVLAALPEFIFYETEELFEETLCSALYPEDTVYSWRHFHTLRMTIFCLVLPLLVMAICYTGIIKTLLRCPSKKKYKAIRLIFVIMAVFFIFWTPYNVAILLSSYQSILFGNDCERSKHLDLVMLVTEVIAYSHCCMNPVIYAFVGERFRKYLRHFFHRHLLM

CCL27:

FLLPPSTACCTQLYRKPLSDKLLRKVIQVELQEADGDCHLQAFVLHLAQRSICIHPQNPSLSQWFEHQERKLHGTLPKLNFGMLRKMG

F-CCL27:

F|FLLPPSTACCTQLYRKPLSDKLLRKVIQVELQEADGDCHLQAFVLHLAQRSICIHPQNPSLSQWFEHQERKLHGTLPKLNFGMLRKMG

NT28-CCL27(9-88):

ILPIASS|CCTQLYRKPLSDKLLRKVIQVELQEADGDCHLQAFVLHLAQRSICIHPQNPSLSQWFEHQERKLHGTLPKLNFGMLRKMG

CCL28(1-105):

ILPIASSCCTEVSHHISRRLLERVNMCRIQRADGDCDLAAVILHVKRRRICVSPHNHTVKQWMKVQAAKKNGKGNVCHRKKHHGKRNSNRAHQGKHETYGHKTPY

CCL28(1-81):

ILPIASSCCTEVSHHISRRLLERVNMCRIQRADGDCDLAAVILHVKRRRICVSPHNHTVKQWMKVQAAKKNGKGNVCHRKK

NT27-CCL28(8-105):

FLLPPSTA|CCTEVSHHISRRLLERVNMCRIQRADGDCDLAAVILHVKRRRICVSPHNHTVKQWMKVQAAKKNGKGNVCHRKKHHGKRNSNRAHQGKHETYGHKTPY

NT27-CCL28(8-81):

FLLPPSTA|CCTEVSHHISRRLLERVNMCRIQRADGDCDLAAVILHVKRRRICVSPHNHTVKQWMKVQAAKKNGKGNVCHRKK

An ensemble of 25 AF2 models (5 random seeds with 5 models per seed) and up to 150 AF3 models were built for each of the following complexes:

● CCR10 w/CCL27
● CCR10 w/F-CCL27
● CCR10 w/NT28-CCL27
● CCR10 w/CCL28
● CCR10 w/NT27-CCL28
● CCR3 w/CCL28
● CCR3 w/NT27-CCL28

Some AF3 models included the C-terminal helix of human Gαi1(GNAI1(327-354), amino acid sequence TDTKNVQFVFDAVTDVIIKNNLKDCGLF), to promote the active state of the receptor TM bundle, and/or explicit sulfotyrosine residues at CCR10 positions Y14 and Y22 or at CCR3 positions Y16 and Y17.

Using ICM software version 3.9-4a (98) the models were superimposed onto a common reference frame and the chemokines were modified to include a free positively charged N terminus (NH_3_^+^). For receptor models built without sulfotyrosines, sulfotyrosines were introduced at this stage. Complexes where the chemokine was not predicted to bind in the orthosteric pocket of the receptor (a common AF2 prediction error for CCR3) were discarded. Chemokine molecules in the remaining complexes were subjected to local gradient minimization with positional harmonic restraints on Cα atoms using internal coordinate mechanics (ICM) (98). Complexes were then scored using the RTCNN, a deep learning–based scoring function implemented in ICM and trained to distinguish protein complexes with potent binders from similar decoy complexes (54, 55, 56, 57, 99). Per-atom RTCNN score contributions were taken from the corresponding neural network layer and averaged across individual residues (for line graphs in Figure 2, Figure 3, Figure 4, Figure 5) or, separately, residue backbones and sidechains (for RTCNN coloring in Figs. 2, 3 and 5). The scores were used to estimate the contributions of the respective residues to the favorable interactions between the receptor and the chemokine.

Models of CCR10 complexes with L-CCL27 were derived from CCR10–F-CCL27 models by mutating Phe0 into Leu0 in ICM. This route was chosen over *ab initio* modeling of CCR10 complexes with L-CCL27 to ensure that the conformation of the chemokine is preserved and the Phe0-*versus*-Leu0 RTCNN comparison is adequate (Fig. 3).

PDB files of all constructed models are provided in Supp. Data 1 (AF2 models) and Supp. Data 2 (AF3 models). Prediction confidence for the entire complex and for the chemokine N terminus in all models is shown in Figs. S1 and S2. For main-text molecular figures, models with close-to-maximum prediction confidence (using AF-provided pLDDT scores) and favorable RTCNN were selected. ICM Browser sessions used for figure generation are provided as Supp. Data 3.

## Data availability

All data needed to evaluate the conclusions in the study are present in the article, supplementary materials, and supplementary data.

## Supporting information

This article contains supporting information.

## Conflict of interest

T. M. H. is a cofounder of Lassogen Inc. and serves on the Scientific Advisory Boards of Abilita Bio, Abalone Bio, and Aikium Inc. The terms of these arrangements have been reviewed and approved by the University of California, San Diego, in accordance with its conflict of interest policies. The other authors declare that they have no conflicts of interest with the contents of this article.
