# Supplementary material for: Rules of engagement: Determinants of chemokine receptor activation and selectivity by CCL27 and CCL28
Source: J Biol Chem. 2025 Sep 18;301(11):110736. doi: 10.1016/j.jbc.2025.110736 (PMC12556802; doi:10.1016/j.jbc.2025.110736)
Supplement: Supporting Materials [file mmc1.docx]

**Title:**

**Rules of engagement: determinants of chemokine receptor activation and selectivity by CCL27 and CCL28**

**Authors:**

Mian Huang^1^, Aura F. Celniker^1, 2^, Rezvan Chitsazi^1, 3^, Douglas P. Dyer^1, 4, 5^, Ariane L. Jansma^1, 6^, Irina Kufareva^1^, Catherina L. Salanga^1^, Tracy M. Handel^1^

**List of materials**

**Figure S1.** 3D model ensembles of receptor-chemokine complexes generated in this work.

**Figure S2.** Per-residue pLDDT scores for the models of receptor-chemokine complexes generated in this work.

**Figure S3.** Calcium flux analysis of CCL27 and F-CCL27.

**Figure S4.** Cell migration analysis of CCL28 N-terminal mutants.

**Figure S5.** Comparison of CCR3 and CCR10 binding pockets.

**Figure S6.** Comparison of electrostatic properties of CCL27 and CCL28.

**Figure S7.** Distinct differences in HS-binding of chemokines and mutants.

**Figure S8.** SPR interaction analysis of chemokines with HS.

**Table S1.** EC_50_ and E_max_ values of CCL27 and mutants in β-arrestin recruitment assays.

**Supp. Data 1**: 3D coordinates of AF2 models of receptor-chemokine complexes presented in the manuscript, in PDB format, with pLDDT scores in the B-factor field.

**Supp. Data 2**: 3D coordinates of AF3 models of the same receptor-chemokine complexes, in PDB format, with pLDDT scores in the B-factor field.

**Supp. Data 3:** Interactive ICM Browser sessions for the molecular figures in the manuscript.


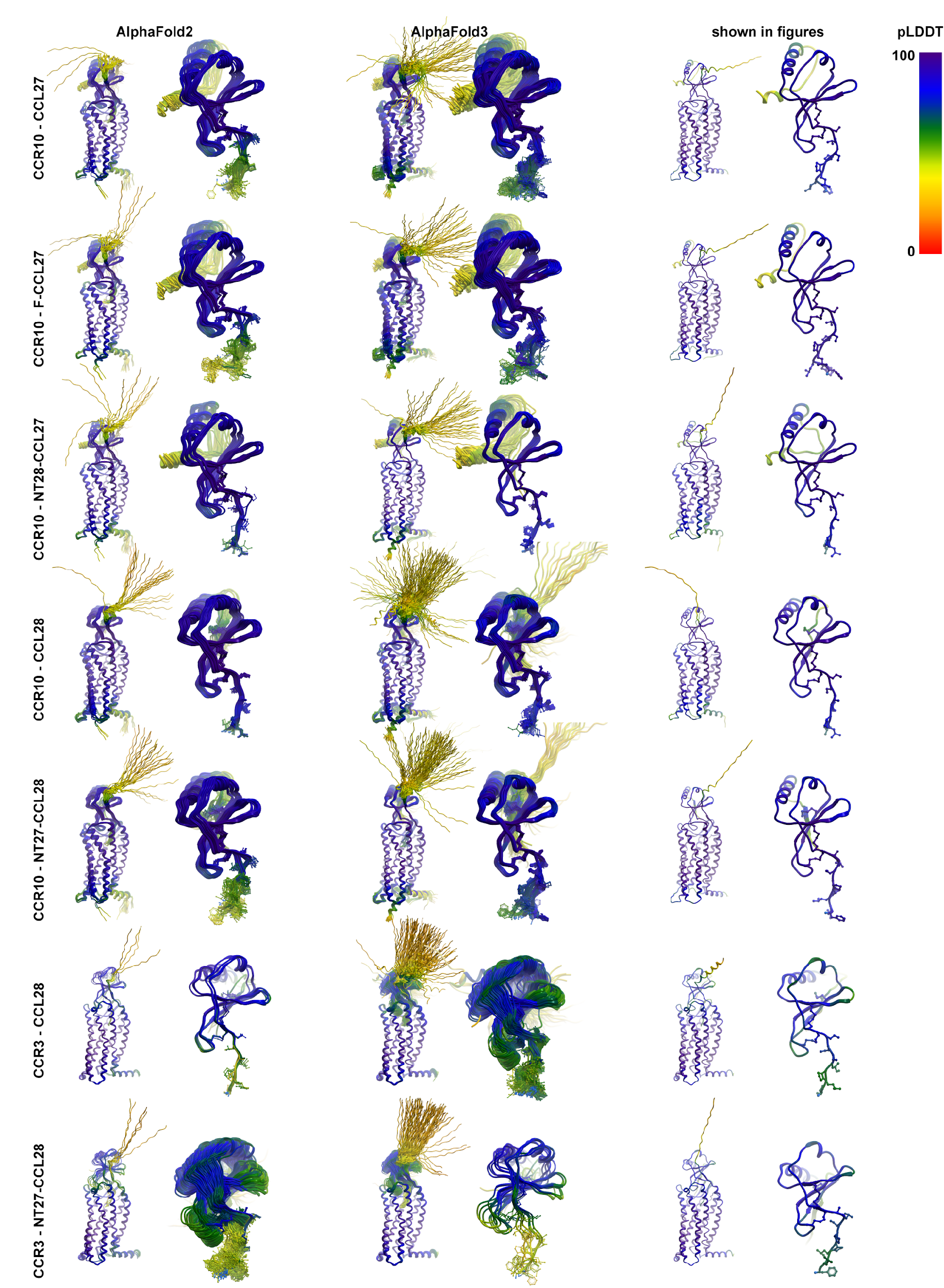


**Figure S1. 3D model ensembles of receptor-chemokine complexes generated in this work.** Each panel shows the model ensemble for the complex on the left and an enlarged view of chemokine molecules in that ensemble on the right. Receptors and chemokines are shown as ribbons and colored by the prediction confidence (pLDDT score) reported by the respective predictor (AF2 or AF3). Rightmost columns present individual models selected for the main-text figures in the manuscript.

**
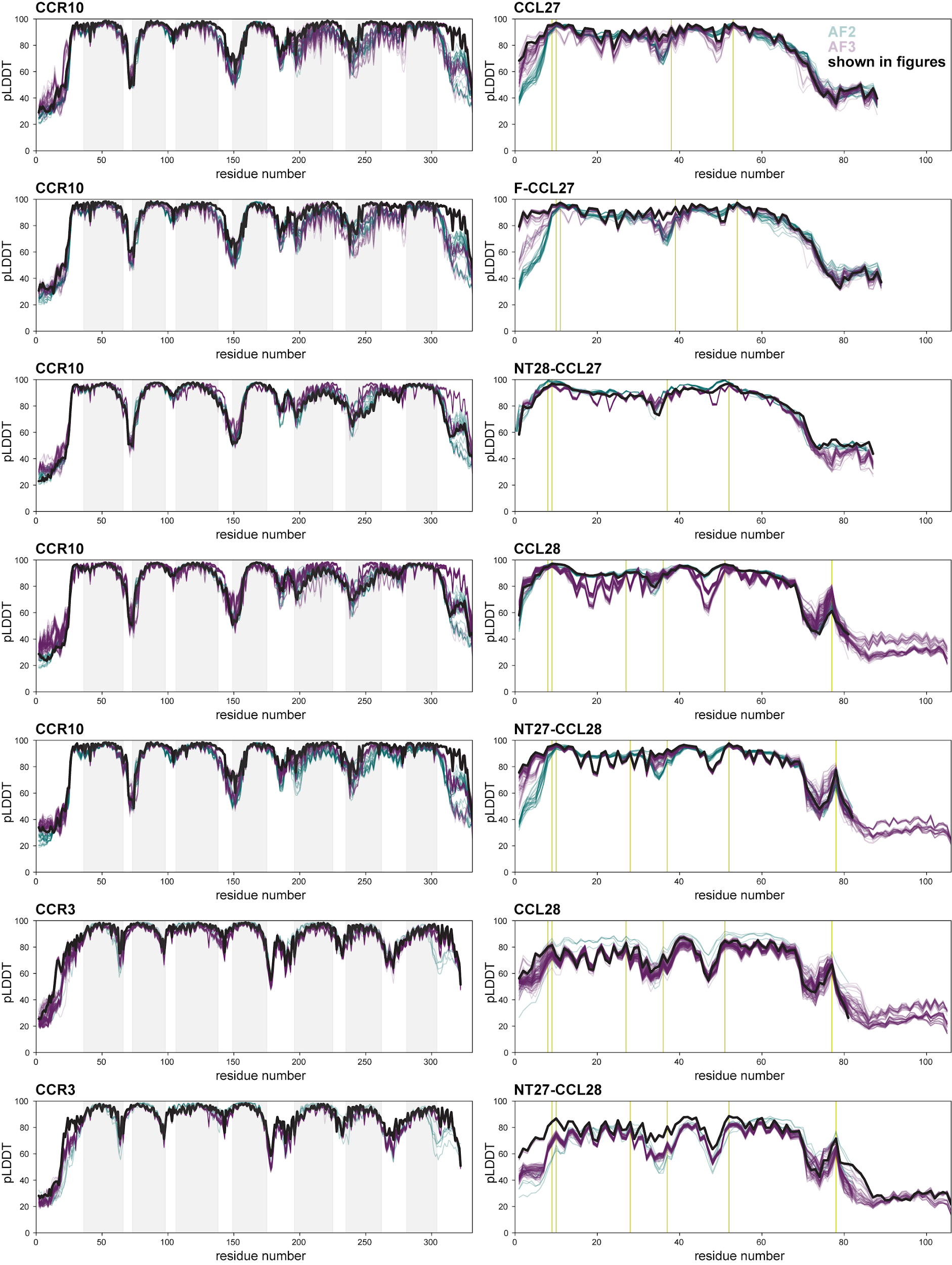
**

**Figure S2. Per-residue pLDDT scores for the models of receptor-chemokine complexes generated in this work.** Scores for the residues in the receptor (left) and chemokine (right) are plotted against amino acid residue number in the respective molecules. Each model is shown as a single trace; models generated by AF2 and AF3 are colored green and purple, respectively; models selected for the main-text figures in the manuscript are shown in bold black trace. Gray rectangles on the left denote the amino acid boundaries for the receptor TM helices. Yellow vertical lines on the right denote the positions of cysteine residues in the chemokines.

**
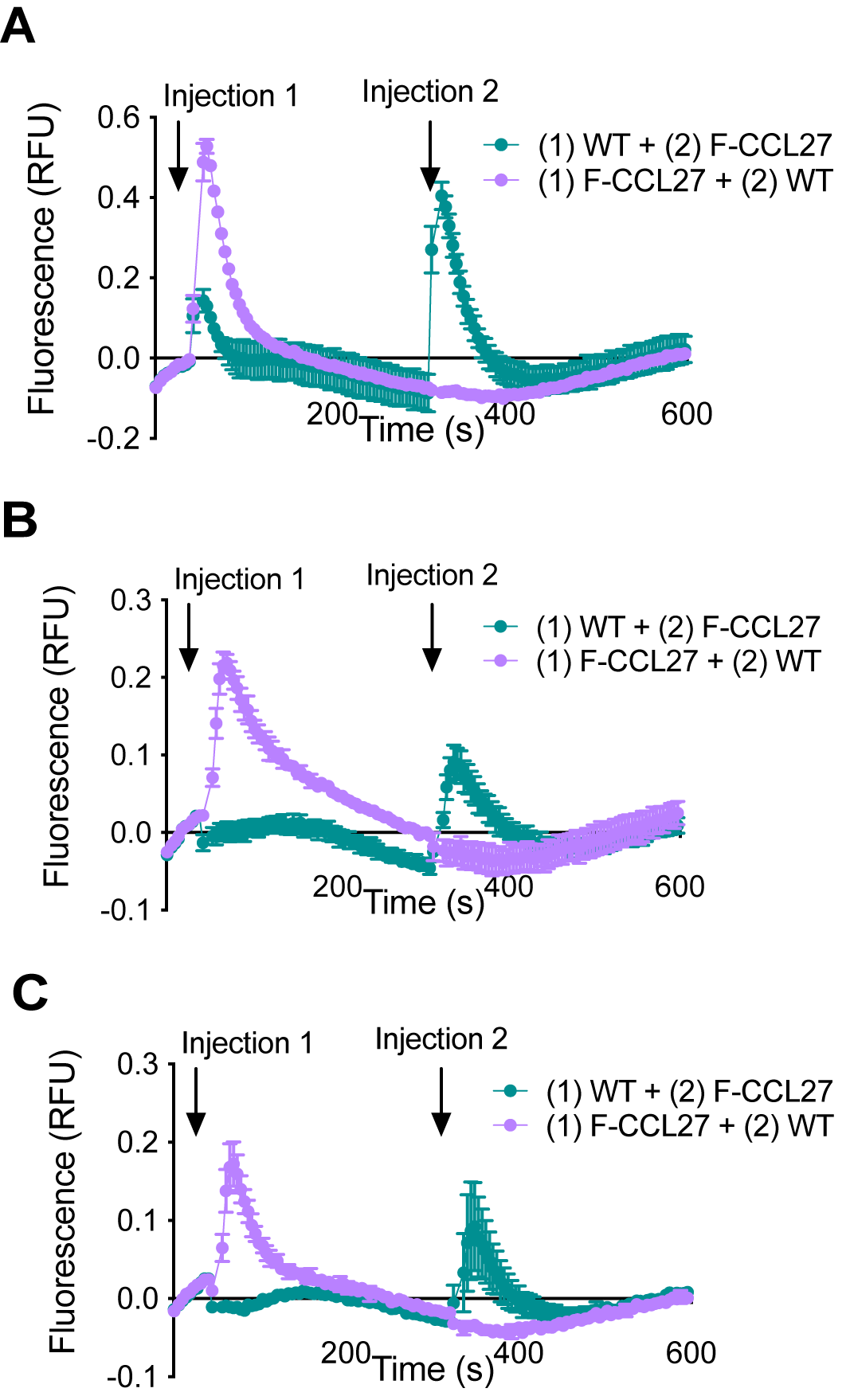
**

**Figure S3. Calcium flux analysis of CCL27 and F-CCL27.**(**A-C**) Chemokine-mediated desensitization of L1.2/CCR10 cells as measured by calcium flux. Cells were treated with an initial dose of 20 nM WT or F-CCL27 (injection 1) followed by a second addition of 20 nM F-CCL27 and WT CCL27 (injection 2), respectively, after the 75th reading cycle (at approximately 320 s). Shown are results from three independent experiments performed in technical triplicates.

**
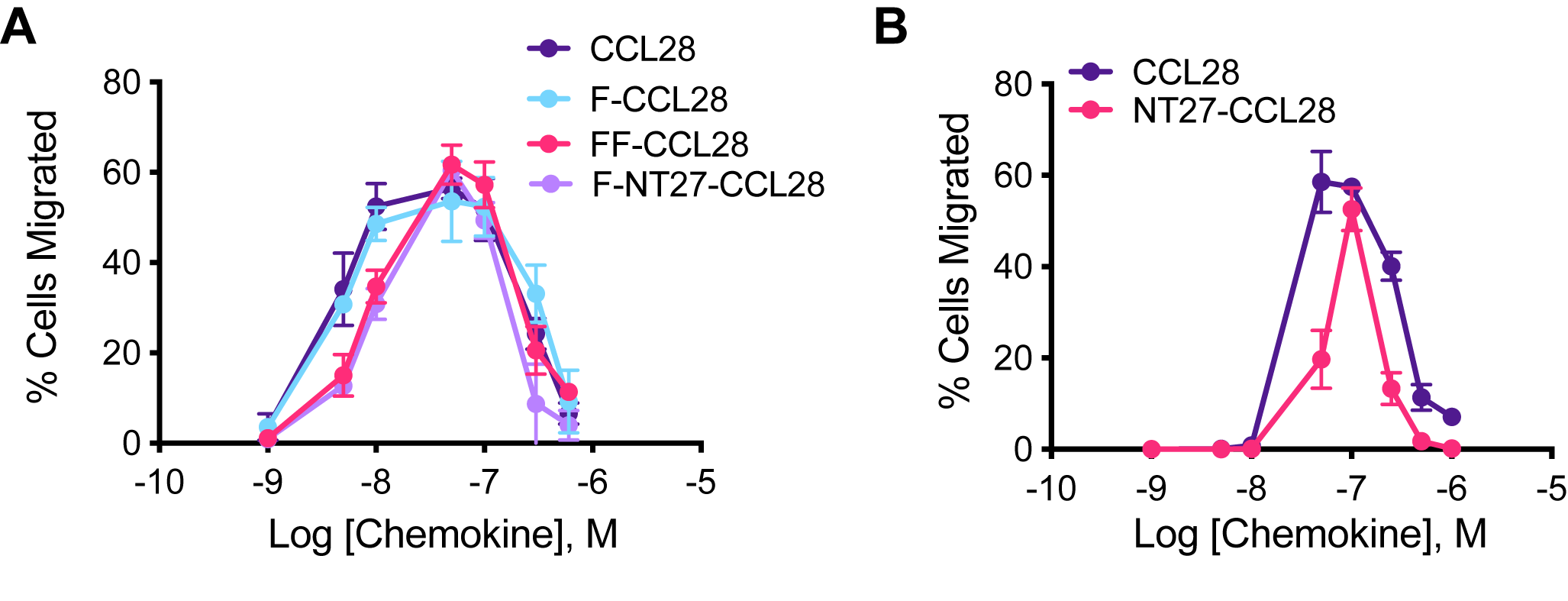
**

**Figure S4. Cell migration of CCL28 N-terminal mutants.** Cell migration with L1.2/CCR10 cells across varying chemokine concentrations of **(A)** CCL28, F-CCL28, FF-CCL28 and F-NT27-CCL28 and **(B)** CCL28 and NT27-CCL28. Data are plotted as the percent of cells migrated after a 2 h incubation at 37°C. Results shown are representative data (mean ± SD) from three independent experiments performed in technical triplicates.

**
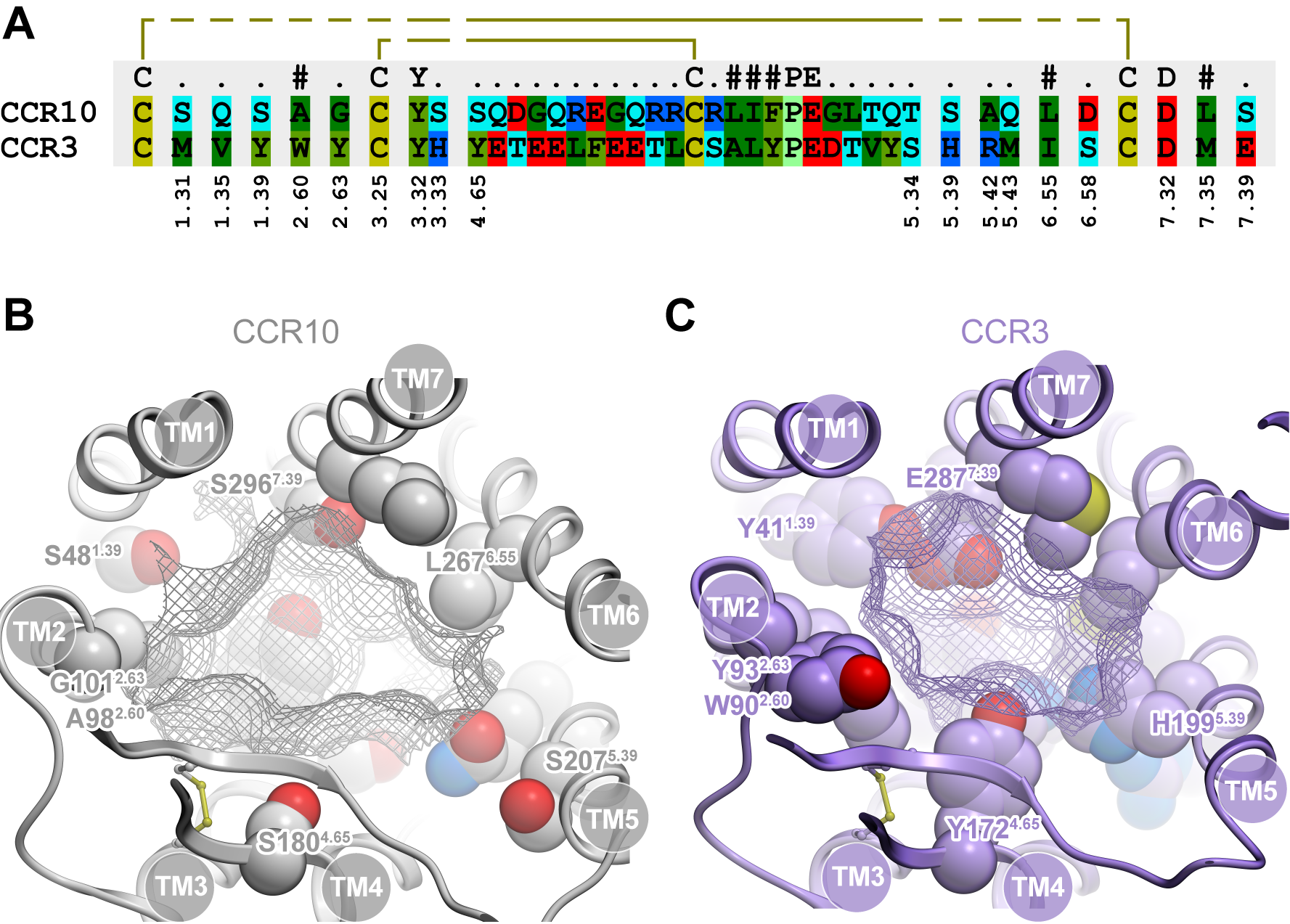
**

**Figure S5.** **Comparison of CCR3 and CCR10 binding pockets.** (**A**) Sequence alignment of pocket residues of CCR3 and CCR10. (**B-C**) Top view of CCR10 (**B**) and CCR3 (**C**) orthosteric binding pockets. The optimal ligand placement surface is shown as a mesh. The amino-acid residues that are not conserved between the two receptors are shown as spheres.


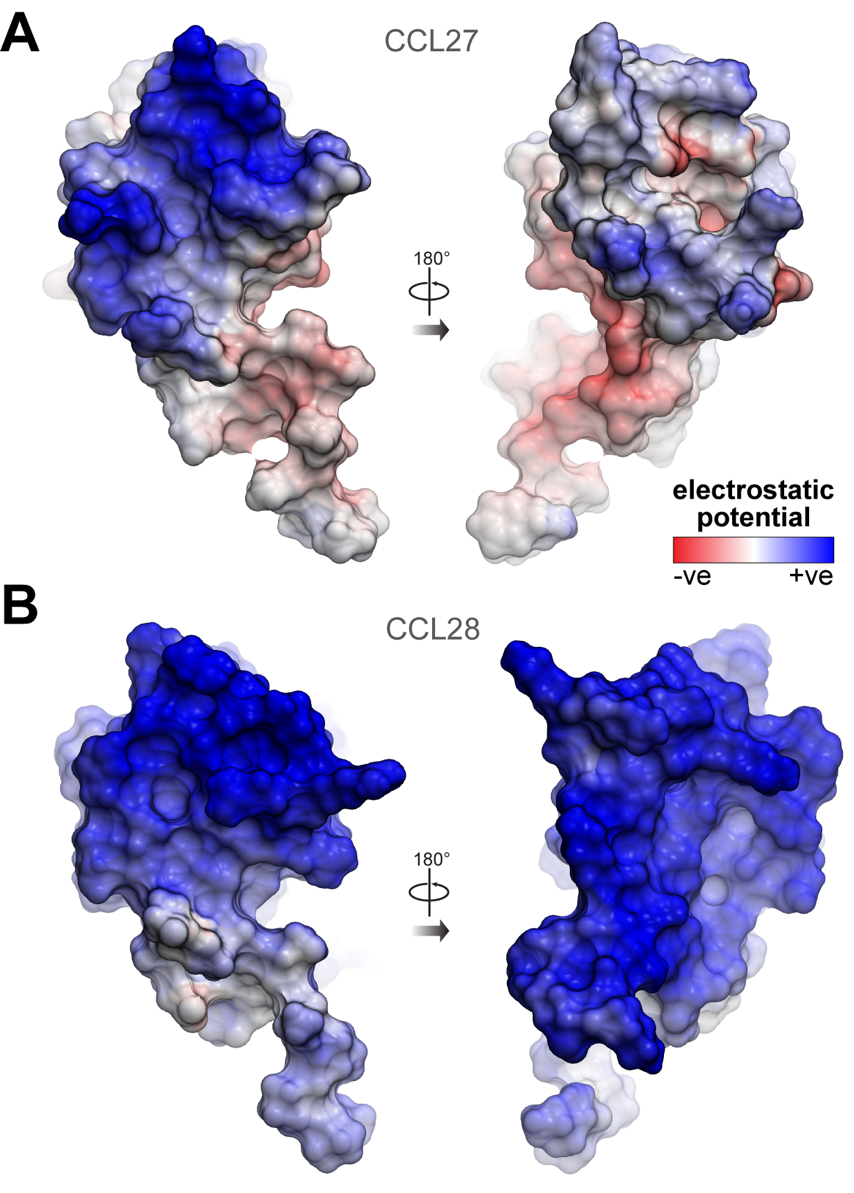


**Figure S6. Comparison of electrostatic properties of CCL27 (A) and CCL28 (B).**

**
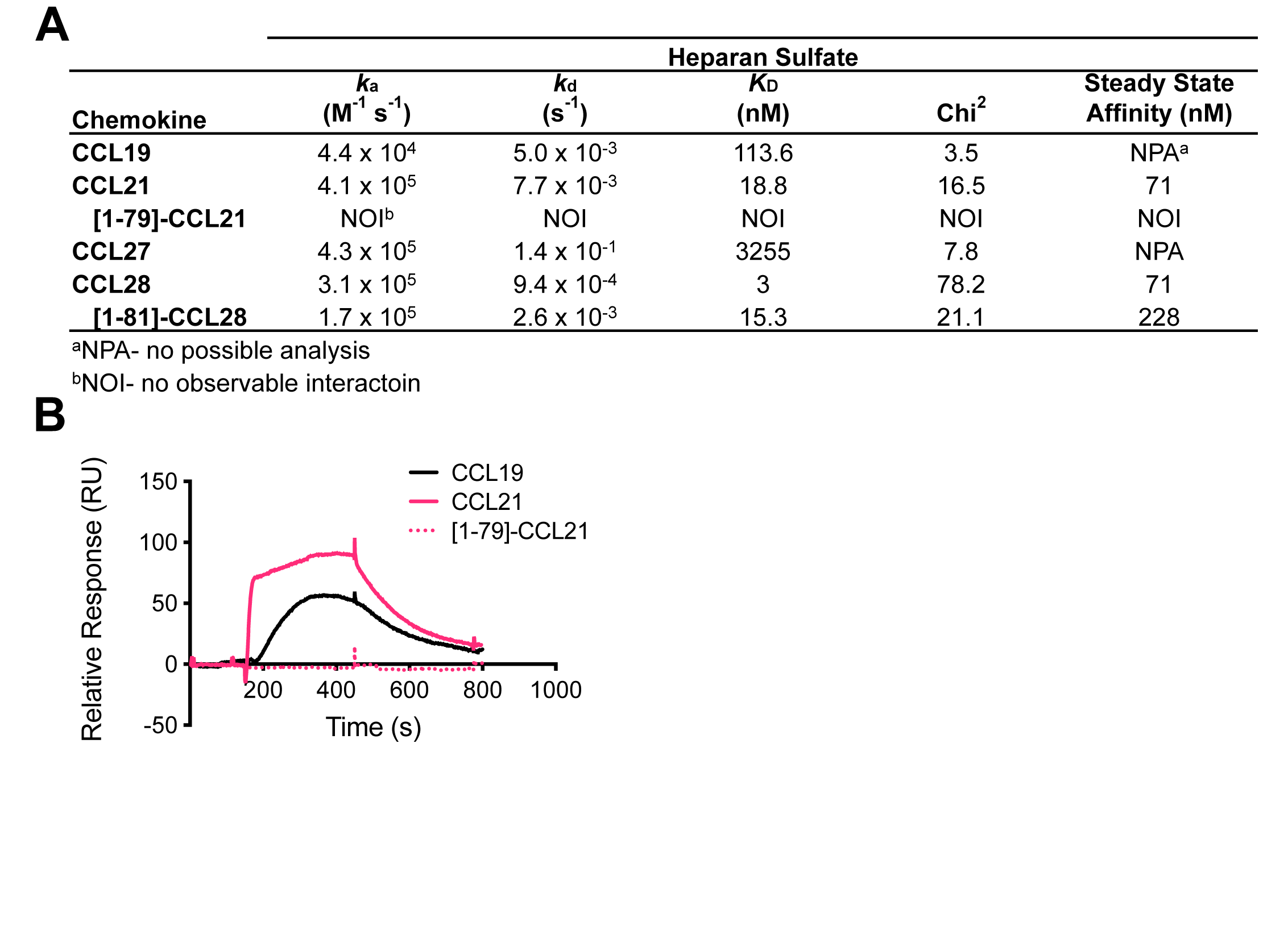
**

**Figure S7**. **Distinct differences in HS-binding of chemokines and mutants.** (**A**) Summary of SPR analysis including the association (*k*_a_), dissociation (*k*_d_) and resulting affinities (*K*_D_=*k*_d_/*k*_a_), and steady state affinity analysis where possible, for chemokine and mutant interactions with HS immobilized on the surface of a C1 chip. Chi^2^ values are included as a measurement of the quality of the data, as described in Experimental Procedures. (**B**) SPR sensorgrams of CCL19, CCL21 and [1-79]-CCL21 showing the resulting signals (relative response in RU) for interaction with immobilized HS with the same concentration of injected chemokine (200 nM).


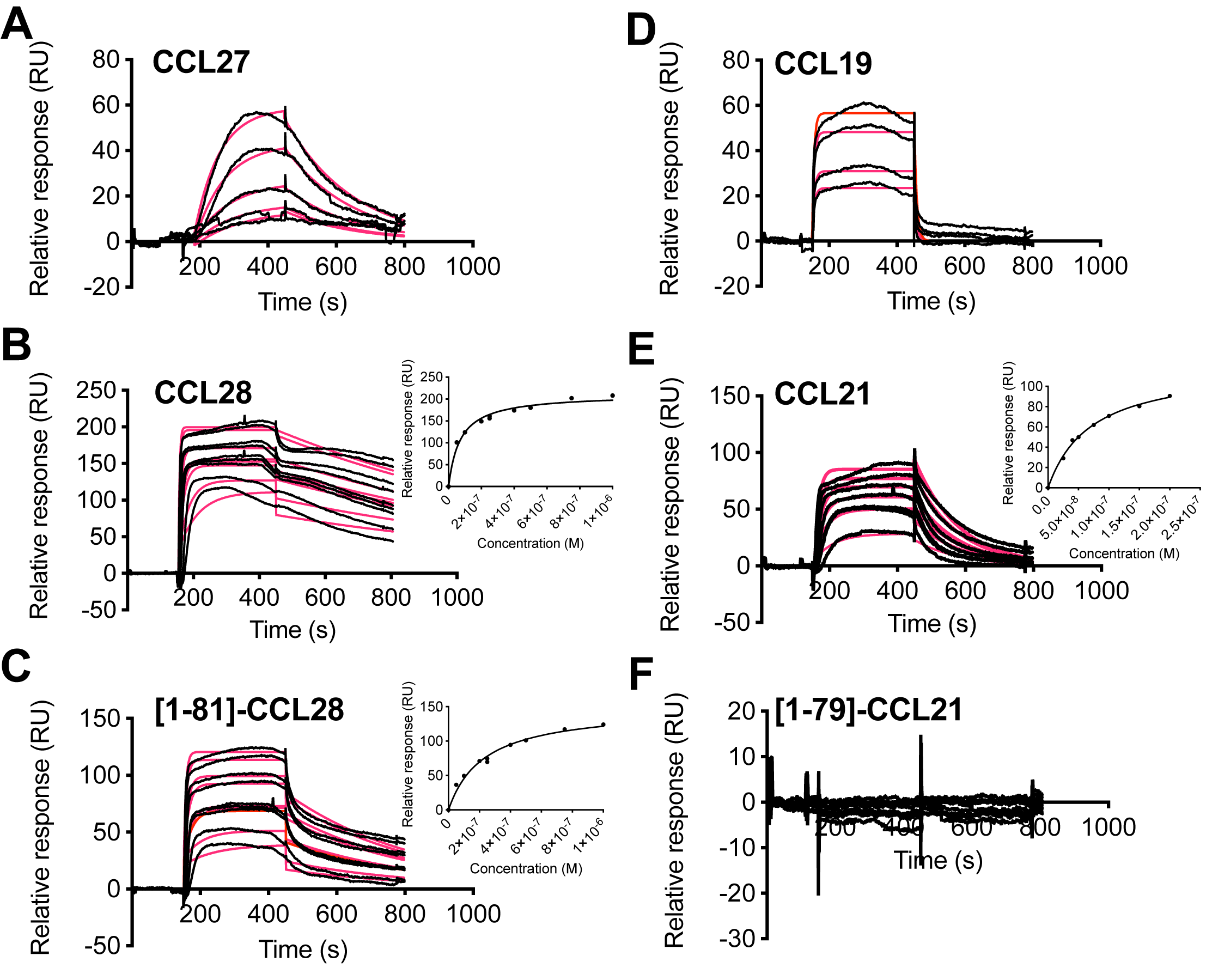


**Figure S8. SPR interaction analysis of chemokines with HS**. (**A**) CCL27 (1000, 750, 500 and 400 nM), (**B**) CCL28 (1000, 750, 500, 400, 250 (repeated), 200, 100 and 50 nM), (**C**) [1-81]-CCL28 (1000, 750, 500, 400, 250 (repeated), 200, 100 and 50 nM), (**D**) CCL19 (200, 150, 100, 75 and 50 nM), (**E**) CCL21 (200, 150, 100, 75, 50, 37.5 and 25 nM) and (**F**) [1-79]-CCL21 (200, 150, 100, 75, 50, 37.5 and 25 nM) with HS immobilized on a C1 chip. Varying concentrations of chemokines were passed over the HS surface and resulting interactions were analyzed using the 1:1 Langmuir model to generate binding affinities (**Supp. Fig. 7A**). Shown are the experimental (black lines) and fitted data (pink lines) against the relative response signal (RUs) for each chemokine concentration series. Where applicable, steady state analysis was also performed; the insets show a plot of the RUs of chemokines at varying concentrations, which was used to determine the binding affinity by steady state analysis.

**Table S1. EC_50_ and E_max_ values of CCL27 and mutants in β-arrestin recruitment assays.** *

|  | **EC_50_ (M)** | **E_max_ (nM)** | **E_max_(mut) / E_max_(WT)** |
| --- | --- | --- | --- |
| **CCL27** | 8.9E-08 ± 2.4E-08 | 101.1 ± 2.9 | 1 |
| **F-CCL27** | 1.9E-08 ± 3.5E-09 | 184.4 ± 2.7 | 1.8 ± 0.1 |
| **NT28-CCL27** | 2.3E-08 ± 5.1E-09 | 152.1 ± 0.3 | 1.5 ± 0.1 |

* EC_50_ and E_max_ values were derived from β-arrestin recruitment assays shown in **Fig. 3D** and **Fig. 4D**. The values were determined by fitting four-parameter logistic regression models to the dose-response curves using GraphPad Prism software version 10.5.0. For each independent experiment, a minimum of two technical replicates were averaged prior to curve-fitting. The EC_50_ and E_max_ values are presented as the mean and standard deviation of a minimum of three independent experiments (n=3 or 5). The last column shows values for the mean and standard deviation of the ratio between E_max_ of the mutant chemokine and the E_max_ of the WT chemokine.

**Supp. Data 1**: 3D coordinates of AF2 models of receptor-chemokine complexes presented in the manuscript, in PDB format, with pLDDT scores in the B-factor field.

**Supp. Data 2**: 3D coordinates of AF3 models of the same receptor-chemokine complexes, in PDB format, with pLDDT scores in the B-factor field.

**Supp. Data 3:** Interactive ICM Browser sessions for the molecular figures in the manuscript.

Supp. Data 1-3 are stored in the URL below:

<https://zenodo.org/records/16102870>
